# Specificity of Nuclear Size Scaling in Frog Erythrocytes

**DOI:** 10.3389/fcell.2022.857862

**Published:** 2022-05-18

**Authors:** Tetsufumi Niide, Saki Asari, Kosuke Kawabata, Yuki Hara

**Affiliations:** Evolutionary Cell Biology Laboratory, Faculty of Science, Yamaguchi University, Yamaguchi, Japan

**Keywords:** nucleus, size scaling, erythrocyte, cell size, genomic content, nuclear size

## Abstract

In eukaryotes, the cell has the ability to modulate the size of the nucleus depending on the surrounding environment, to enable nuclear functions such as DNA replication and transcription. From previous analyses of nuclear size scaling in various cell types and species, it has been found that eukaryotic cells have a conserved scaling rule, in which the nuclear size correlates with both cell size and genomic content. However, there are few studies that have focused on a certain cell type and systematically analyzed the size scaling properties in individual species (intra-species) and among species (inter-species), and thus, the difference in the scaling rules among cell types and species is not well understood. In the present study, we analyzed the size scaling relationship among three parameters, nuclear size, cell size, and genomic content, in our measured datasets of terminally differentiated erythrocytes of five Anura frogs and collected datasets of different species classes from published papers. In the datasets of isolated erythrocytes from individual frogs, we found a very weak correlation between the measured nuclear and cell cross-sectional areas. Within the erythrocytes of individual species, the correlation of the nuclear area with the cell area showed a very low hypoallometric relationship, in which the relative nuclear size decreased when the cell size increased. These scaling trends in intra-species erythrocytes are not comparable to the known general correlation in other cell types. When comparing parameters across species, the nuclear areas correlated with both cell areas and genomic contents among the five frogs and the collected datasets in each species class. However, the contribution of genomic content to nuclear size determination was smaller than that of the cell area in all species classes. In particular, the estimated degree of the contribution of genomic content was greater in the amphibian class than in other classes. Together with our imaging analysis of structural components in nuclear membranes, we hypothesized that the observed specific features in nuclear size scaling are achieved by the weak interaction of the chromatin with the nuclear membrane seen in frog erythrocytes.

## Introduction

Eukaryotic cells have a nucleus for carrying out genomic functions, such as DNA replication and transcription. The size of the nucleus changes dynamically during the differentiation and development of eukaryotes. For instance, during metazoan embryogenesis, the nuclear size decreases as the cell (blastomere) size reduces by half every cell division, without cell growth (Wesley et al., 2020). Changes in the nuclear size coincide with changes in cellular functions related to not only genetic functions such as transcription and chromosome condensation (Hara et al., 2013; Jevtić and Levy, 2015) but also non-genetic functions such as cell cycle control and cell migration (Nolet et al., 2020; Rizzotto et al., 2020). Therefore, the cell is required to organize the nuclear size to suitably fit nuclear functions to cellular environments. In line with this, cell size, one of the key cellular parameters reflecting the environment, has long been known to scale to the nuclear size over 100 years (Conklin, 1912). Aberrant scaling of nuclear size to cell size has frequently been observed in multiple cancer types and correlates with metastasis (Zink et al., 2004; de Las Heras and Schirmer, 2014), suggesting that nuclear size scaling is one of the fundamental intracellular rules for organizing cellular and genetic functions.

In various eukaryotes, a correlation has been observed between the nuclear and cell sizes. In individual unicellular species or specific cell types of multicellular eukaryotes, the nuclear volume is almost proportional to the cellular volume, over spontaneous changes in the cellular volume, such as in yeasts (Jorgensen et al., 2007; Neumann and Nurse, 2007), embryonic cells in frogs (Jevtić and Levy, 2015), and culture cells in human (Viana et al., 2020), etc., This proportionality can be converted to a constant ratio of the nuclear volume to the cell volume (N/C volume ratio). In contrast, when analyzing the scaling of the nuclear size to the cell size among non-biased cell types from various species, the nuclear volume does not reveal a strict proportional relationship and constant N/C volume ratio, thus revealing an allometric scaling relationship (Hara, 2020; Malerba and Marshall, 2021). To analyze this allometry, the relationship between nuclear and cell sizes is generally expressed as *NV ∼ CV* ^*A*^ where *NV*, *CV*, and *A* are the nuclear volume, cell volume, and scaling exponent, respectively. The calculated scaling exponent *A* in each species group of multicellular eukaryotes, unicellular eukaryotes, or prokaryotes (which have the nucleoid) is usually in the range of 0.8–0.9, indicating hypoallometry (Hara, 2020; Malerba and Marshall, 2021). Nonetheless, there are some variations in the *A* value, such as a remarkably low value (*A* ≅ 0.4) in intestinal cells of worms (Uppaluri et al., 2016). Furthermore, even in cell types showing an isometric relationship with *A* ≅ 1, the absolute value of N/C volume ratio shows variation among species and cell types, such as 0.08–0.10 in yeasts (Jorgensen et al., 2007; Neumann and Nurse, 2007), ∼0.04 in embryonic cells of frogs (Jevtić and Levy, 2015), and ∼0.2 in some angiosperms (Milo and Philips, 2016). Despite “quantitative” variations in values of the scaling exponent *A* and N/C volume ratio, the “qualitative” scaling relationship of nuclear size with cell size is well conserved among eukaryotes and prokaryotes having nucleoids (Hara, 2020; Malerba and Marshall, 2021). Mechanistic insights underlying the size scaling properties of some model species have been partially elucidated. Nuclear size is controlled by the ability to supply nuclear constituents from the cytoplasm to the nucleus, such as lamin proteins, which are composed of nuclear lamina underneath the nuclear envelope and are selectively imported through nuclear pore complexes (NPCs) (Levy and Heald, 2010; Jevtić et al., 2015), and lipid membranes, which are supplied from the cytoplasm to the nuclear membrane through the endoplasmic reticulum (ER) (Hara and Merten, 2015; Kume et al., 2019; Mukherjee et al., 2020). Assuming that the quantity of these determinants for the nuclear size is finite in the cytoplasm and proportional to cytoplasmic volume, the proportional scaling relationship between nuclear and cell sizes could be explained (Goehring and Hyman, 2012).

Apart from its scaling relationship with cell size, the nuclear size is also known to correlate with the genomic content inside the nucleus. Establishment of this correlation has been carried out using inter-species datasets of specific cell types (Price et al., 1973; Olmo and Morescalchi, 1978; Cavalier-Smith, 1985; Gregory, 2001b) and intra-species datasets of cells with different ploidy levels (Gillooly et al., 2015; Robinson et al., 2018). Recent *in vitro* cell-free reconstruction systems from *Xenopus* egg extract have indicated that experimental manipulation of DNA content inside the nucleus could change the nuclear expansion dynamics in interphase, without changing the extract volume (Heijo et al., 2020). Furthermore, modification of the condensation state alters the nuclear size without manipulating the DNA content in isolated nuclei and the cell-free system (Chan et al., 2017; Shimamoto et al., 2017; Heijo et al., 2020). Together with the experimental evidence, it can be concluded that the chromatin inside the nucleus plays a non-negligible role in controlling nuclear size. Nonetheless, *in vivo*, the cell size also generally correlates with the genomic content in various cell types of individual eukaryotic species and among species (Gregory, 2001a; Gillooly et al., 2015; Levy and Heald, 2015). Furthermore, in individual species, cell size generally varies among cell types, without changing the genomic content. Therefore, the observed stronger correlation of the cell size with the nuclear size and that of the genomic content with the cell size might mask the contribution of the genomic content in the determination of nuclear size. These cross-correlations among the three parameters of cell size, nuclear size, and genomic content make it difficult to discuss the contribution of one parameter to the other.

Previous inter-species analyses concerning nuclear size scaling have utilized datasets from non-biased cell types of various species and averaged the values from each species (Hara, 2020; Malerba and Marshall, 2021). In particular, very few studies have systematically analyzed the specificities of the nuclear size scaling in each cell type and compared intra-species scaling features with inter-species features. The lack of this knowledge prevents us from understanding the comprehensive mechanisms underlying nuclear size scaling and gain an evolutionary view of how the nucleus and cell have co-evolved, accompanied by changes in the genomic contents. In the present study, we compiled datasets of nuclear size, cell size, and genomic content by measuring erythrocytes from five different frogs, including clawed frogs, *Xenopus laevis*, *Xenopus tropicalis*, and Japanese regional frogs *Hyla japonica*, *Glandirana rugosa*, and *Fejervarya kawamurai*, and our collection of erythrocytes across species classes from published studies. Using these datasets that focus on a single cell type among species, we compared intra-species features of nuclear size scaling with inter-species features in different frogs and in each species class. Based on the comparison, we identified the specificities of scaling of nuclear size to cell size and genomic content, in erythrocytes and in each species class.

## Materials and Methods

### Animals

*X. laevis* adults were purchased from a local breeder (Watanabe Zoshoku, Hyogo, Japan). Fertilized eggs were obtained by means of insemination and fed until they developed into larvae or young adults. *X. tropicalis* was provided by Amphibian Research Center (Hiroshima University) through the National Bio-Resource Project of MEXT, Japan. *H. japonica*, *G. rugosa*, and *F. kawamurai* adults were caught in a rice field near Yamaguchi University.

### Cellular Sample Collection

Blood samples were collected from the hearts of anesthetized frogs after injecting the animals with 0.2% MS222 (ethyl 3-aminobenzoate methanesulfonate; catalog no. E1052, Sigma-Aldrich) in 100% De Bore’s solution [110 mM NaCl, 1.3 mM KCl, 5.7 mM Tris, pH 7.5 (NaOH)] with 5 mg/ml ethylenediamine-N,N,N′,N′-tetraacetic acid (EDTA). In case of larva, the body near the heart was cut using tweezers and the blood samples were collected by means of pipetting with 5 μl of 0.8 × phosphate-buffered saline [1 × PBS; 8.04 mM Na_2_HPO_4_, 1.763 mM KH_2_PO_4_, 136.8 mM NaCl, 268 μM KCl, pH 7.4 (NaOH)]. The collected solution was transferred to 100% De Bore’s solution with 5 mg/ml EDTA. The solution was centrifuged at 300 × *g*, 4°C for 15 min. The pellet was resuspended in 0.8 × PBS and centrifuged at 1,000 × *g*, 4°C for 15 min. This washing step was repeated twice. The final pellet was resuspended in a designated volume (almost the same as the collected blood volume) of 0.8 × PBS.

For tissue samples, pieces of liver or kidney were dissected from anesthetized *X. laevis* adult frogs and homogenized in 0.8 × PBS using a micro-tube pestle. These suspensions were used for further experiments. For embryonic samples, fertilized eggs were obtained by *in vitro* insemination of freshly laid *X. laevis* eggs with crushed *X. laevis* testes. Thirty minutes after insemination, the embryos were dejellyed with 2% cysteine [pH 7.8 (KOH)] and washed with 0.2 × MMR [1 × MMR: 0.25 mM 4-(2-hydroxyethyl)-1-piperazineethanesulfonic acid (HEPES)-KOH, 0.1 mM EDTA, 5 mM NaCl, 2 mM KCl, 1 mM MgCl_2_, 2 mM CaCl_2_, pH 7.8] 3–5 times. The embryos were incubated in 0.2 × MMR at 18°C for ∼8.5 days so that the developmental stages of the swimming tadpole embryos could be confirmed. Then, the embryos were homogenized in 0.8 × PBS using a micro-tube pestle. These suspensions were used for further experiments.

### Staining and Observation

For observation of erythrocytes, the prepared erythrocyte solution was mixed with the same volume of a solution containing 4% formaldehyde and 15% glycerol with 5 μg/ml Hoechst 33342 (catalog no. H1399, Invitrogen), and 10 μg/ml 3,3′-dihexyloxacarbocyanine iodide [DiOC_6_(3), catalog no. 318426, Sigma-Aldrich]. Approximately 6 μl of the mixture was placed on a glass slide and covered with a coverslip. The observation slide was subjected to fluorescence imaging using an Eclipse Ti-E (Nikon) wide-field microscope equipped with a ×40 objective lens and an sCMOS camera Zyla4.2 (Andor) motorized by Micro-Manager. To measure the three-dimensional volumes of erythrocytes and nuclei, the slides were subjected to z-sectional imaging using a FV1200 confocal microscope (Olympus) equipped with a ×60 objective lens. For immunostaining of erythrocytes and tissue nuclei, 50 μl of the prepared erythrocyte and tissue or embryo samples were gently mixed with 1 ml of 30% glycerol and 5% formaldehyde. After incubation for 15 min at 23°C, the fixed fractions were sedimented on poly L-lysine-coated coverslips by means of centrifugation at 300 × *g* and 16°C for 15 min. The coverslips were subsequently fixed in ice-cold methanol for at least 15 min, at −20°C. After washing with 1 × PBS, the coverslips were incubated in blocking solution (PBS containing 2% bovine serum albumin and 0.1% Triton™ X-100) for at least 1 h. The coverslips were subsequently treated with anti-nucleoporin antibodies (clone: mab414; 500 × dilution, BioLegend) or anti-pan lamin antibodies (catalog no. 65147; 50 × dilution, PROGEN Biotechnik) for 1 h at 23°C. Anti-pan lamin antibodies are multi-epitope cocktails that recognize multiple lamin isotypes (LA, LI, LII, LIII, B1, B2, and C) expressed in *X. laevis*. Antibodies for nucleoporins can recognize conserved domain FXFG repeats in nucleoporins. Because the NPCs consist of multiple nucleoporins, including FXFG repeats, the antibodies were expected to interact with all NPCs. After washing with PBS containing 0.1% Tween-20 three times, the coverslips were subsequently treated with Alexa Fluor™ 633 anti-mouse IgG antibody (catalog no. A11003; 1,000 × dilution; Thermo Fisher Scientific) for 1 h at 23°C. Finally, the coverslips were stained with 10 μg/ml Hoechst 33342 and 10 μg/ml DiOC_6_(3) for 10 min at 23°C. The stained coverslips were subjected to z-sectional imaging using the above-mentioned wide-field microscope equipped with a ×40 objective lens, and deconvolution was performed using the Microvolution ImageJ plugin (Microvolution, CA, United States).

### Measurements and Statistical Analyses

The cross-sectional area of the Hoechst 33342-stained nuclei was measured using elliptical ROI sections in the ImageJ software (National Institutes of Health). The elliptical ROI was set on the rim of the Hoechst 33342-positive chromatin region. To measure the erythrocytic areas, the cross-sectional area of erythrocytes in phase contrast images was measured using the elliptical sections tool of the ImageJ software. To measure the shape parameters in erythrocytes, the lengths along a long axis, defined as *length*, and along a short axis (perpendicular to the long axis), defined as *width*, were measured using a straight-line ROI in the ImageJ software. The aspect ratio and contour length of the erythrocytes were calculated using formulas as *(length)/(width)* and *2 × π × [(length/2)* ^*2*^ *+ (width/2)* ^*2*^ *]* ^*0.5*^, respectively. The shape index was calculated using the formula as *(contour length)/[π × (length/2) × (width/2)]*. We measured at least 150 erythrocytes from individual frogs, and calculated the mean value for each frog. The thickness of the disc-shaped erythrocytes was measured directly, with the erythrocytes standing vertically inside the sample slide. Ordinary least-squares regression was performed to generate a regression line and calculate the correlation coefficient (*r*) and coefficient of determination (*R* ^*2*^) using the regression analysis in the Excel software (Microsoft). To measure the volumes of erythrocytes and nuclei, the z-sectional images made using DiOC_6_(3) and Hoechst 33342 were subjected to three-dimensional reconstruction, and their volumes and surface areas were determined using the IMARIS software (Oxford Instruments). For quantification of immunostaining signal intensities, the images were acquired under the same exposure conditions as the samples that they were compared with. ROIs were selected by tracing the nucleus rim using the segmented line tool of ImageJ. The mean intensity per pixel on the line ROIs was measured, followed by subtraction of the background signal from it. To normalize the signal intensity between erythrocyte and tissue or embryo samples, the calculated intensities in each erythrocyte nucleus and liver nucleus were divided by the mean intensity of erythrocyte nuclei from individual frogs. Significant differences in erythrocyte and nuclear size parameters, and signal intensities among samples were determined by non-parametric Wilcoxon tests using the R software.

### Data Collection

For comparison of size scaling parameters among wider species classes, we collected the data of erythrocytic and nuclear sizes from the Cell Size Database (http://www.genomesize.com/cellsize/) and published papers. In cases where the lengths of the long (*length*) and short axes (*width*) were previously described in the literature, the cross-sectional area was calculated using the formula, *π × (length/2) × (width/2)*. When only images were shown in the papers, we measured the nuclear and erythrocytic cross-sectional areas using the above-mentioned methods from the published images and then calibrated the absolute area to the scale bar. The genomic content was estimated based on the C-value in the Animal Genome Size Database (http://www.genomesize.com/). All values used in this study are listed in the Supplementary Tables.

## Results

### Nuclear Size Correlates Weakly With Erythrocytic Size in Individual Frogs

**FIGURE 1:**
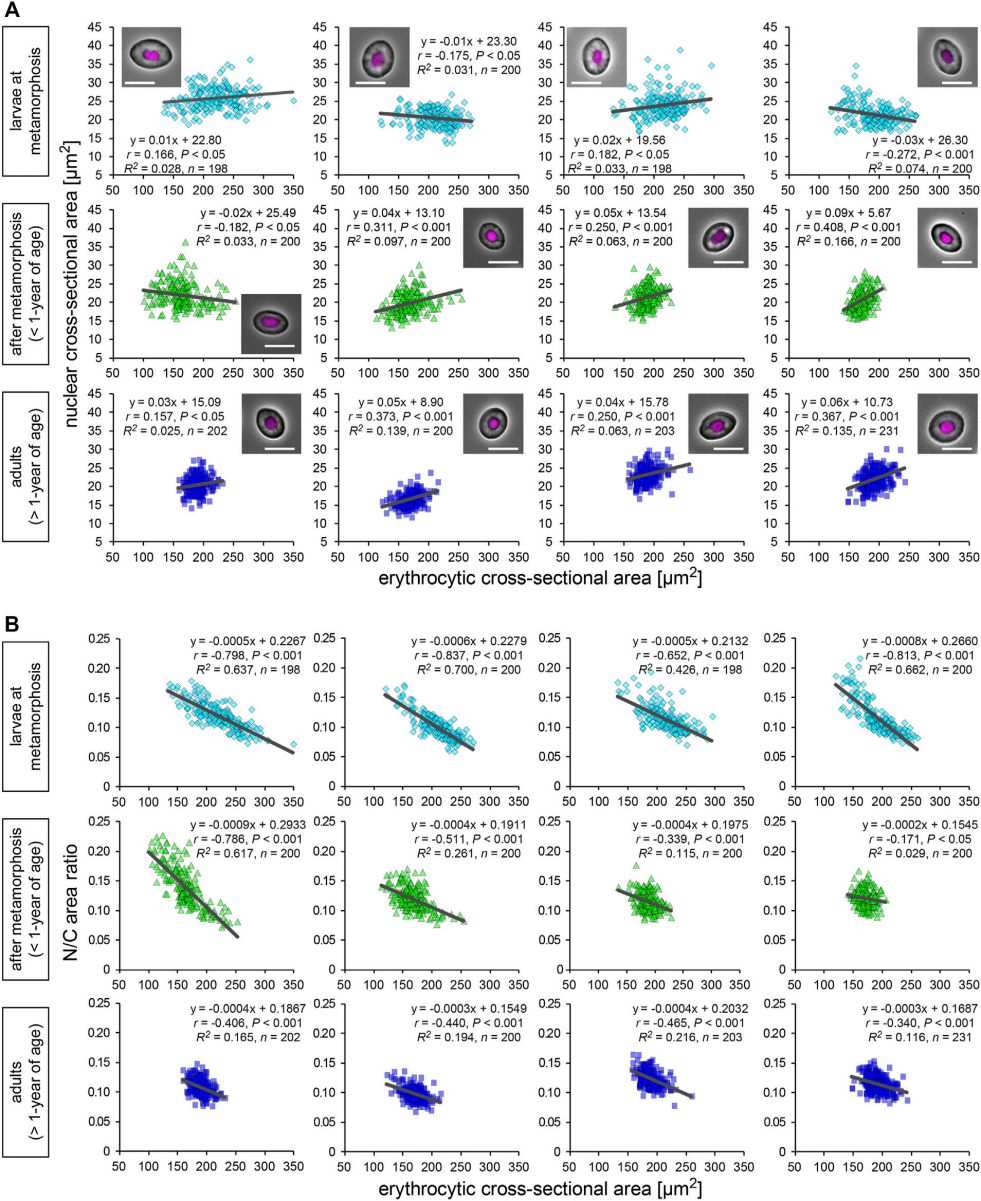
Nuclear and erythrocytic cross-sectional areas from *X. laevis* individuals. **(A)** Measured nuclear cross-sectional areas were plotted against the erythrocytic cross-sectional areas. Each symbol corresponds to data from each erythrocyte. The plots show the datasets from *X. laevis* individuals at the stages of larvae during metamorphosis (light blue), young adults (green), and fully grown adults (blue). A merged image of an erythrocyte with Hoechst 33342 (magenta) staining and phase contrast is shown in each graph. Scale bar = 10 μm. The equation for the regression line, sample number (*n*), Pearson’s correlation (*r*), *P* values, and the coefficient of determination (*R* ^*2*^) have been indicated in each plot. **(B)** Calculated ratios of nuclear cross-sectional area to erythrocytic area (N/C area ratio) have been plotted against the measured erythrocytic areas.

We isolated erythrocytes from individual *X. laevis* adult frogs and measured the cross-sectional areas of the nuclei and whole erythrocytes (Figure 1A). Erythrocytic size is known to vary in different environments such as nutrient conditions (Maekawa et al., 2012; Janiga et al., 2017) and during metamorphosis and larval development (Hota et al., 2013). To collect samples of different sizes, we isolated erythrocytes from different developmental stages, larvae at metamorphosis stages, young adults after metamorphosis (less than 1-year of age), fully grown adults (more than 1-year of age). In the larval samples, the measured mean values and the coefficient of variation (CV) of erythrocytic areas [mean (±SD): 211 ± 20 μm^2^; CV (±SD): 14.5 ± 1.5%] were significantly higher than those in fully grown adults (179 ± 12 μm^2^; 9.8 ± 2.2%) and young adults (170 ± 13 μm^2^; 10.6 ± 3.1%) (Supplementary Table S1; Supplementary Figure S1A). Furthermore, when the lengths of erythrocytes along the long (*length*) and short axes (*width*) were measured, the aspect ratio [(*width*)/(*length*)] of the erythrocytes was not significantly different when these developmental groups were compared (Supplementary Table S1; Supplementary Figure S1B). Although variation in the aspect ratio was detected among individual frogs, there were fewer differences in erythrocytic shape during development. Considering that the conversion of larval erythrocytes to adult erythrocytes occurs after metamorphosis (Tamori and Wakahara, 2000), the observed changes in the mean erythrocytic area may be caused by the conversion of erythrocytes during development. Furthermore, when the volumes of erythrocytes isolated from a single adult frog were estimated using three-dimensional reconstruction, the measured cross-sectional areas were almost proportional to the estimated volumes (Supplementary Figure S1D). We also detected fewer differences in erythrocyte heights through direct measurement among samples from different adult frogs (Supplementary Figure S1E). Therefore, our measurements for two-dimensional sectional areas of the erythrocytes revealed almost similar results for the three-dimensional volume. It should be noted that even when processing the automatic reconstruction of erythrocyte volume, variations in the measured volumes were still observed (Supplementary Figure S1D). Therefore, the measured variation in erythrocyte size was mainly caused by the intrinsic variation of the erythrocytes rather than measurement noise. The mean value of the measured nuclear area was also significantly larger in erythrocytes from larvae [mean (±SD): 23.4 ± 2.5 μm^2^] than those from fully grown adults (22.1 ± 2.5 μm^2^) and young adults (21.4 ± 1.5 μm^2^) (Supplementary Figure S1C). In addition to the erythrocytic shape, the estimated three-dimensional volumes of the nucleus were plotted against the measured nuclear cross-sectional areas (Supplementary Figure S1F). Although a coefficient of determination *R* ^*2*^ *,* which is the ratio of the explained variation from the regression line, is smaller in nuclear parameters than that in erythrocytic parameters, we detected a significant proportionality between nuclear area and volume. This result suggested that the nuclear cross-sectional areas revealed similar tendencies for the nuclear volume. When comparing the nuclear areas with the erythrocytic areas in the datasets from individual frogs were compared, very weak (correlation coefficient *r* < 0.4) but statistically significant (*p* < 0.05) correlations were observed in some samples of all three developmental stages [mean correlation coefficient *r* (±SD) = 0.22 ± 0.16 in fully grown adults; 0.14 ± 0.13 in young adults; 0.02 ± 0.10 in larvae; Figure 1A]. Although the significant correlations were detected in some samples, the slope values of the regression lines were very small in all samples [mean slope (±SD) = 0.20 ± 0.29 in fully grown adults; 0.03 ± 0.03 in young adults; −0.00 ± 0.01 in larvae]. Together with the very small coefficient of determination *R* ^*2*^ in each frog (0.06 ± 0.05 in fully grown adults; 0.04 ± 0.02 in young adults; 0.02 ± 0.04 in larvae; Figure 1A), the measured nuclear areas did not change significantly when the erythrocytic areas increased. Furthermore, an analysis of the ratio of nuclear area-to-erythrocytic (cell) area (N/C area ratio) showed a negative correlation with the erythrocytic area in all three categories (*r* = −0.39 ± 0.18 in fully grown adults; −0.51 ± 0.07 in young adults; −0.76 ± 0.21 in larvae; Figure 1B). This negative correlation could be mathematically explained by the observed less correlation between the nuclear area and the erythrocytic area. Next, to evaluate the known effects of cell surface area and the ratio of surface area to cell volume to control the nuclear size (Brownlee and Heald, 2019), we plotted the measured nuclear areas with the calculated contour lengths of the erythrocyte sections or the shape index, which is a ratio of the contour length to the cell area (Supplementary Figure S2A). However, these two parameters showed a very weak correlation with nuclear area in most samples from each developmental stage (Supplementary Figure S2A). Furthermore, the nuclear area showed no significant correlation with the estimated volume and surface area of erythrocytes from three-dimensional reconstruction (Supplementary Figure S2B). Collectively, the observed less correlation of the nuclear area with any cell size parameter in *X. laevis* erythrocytes is distinct from the previously observed apparent correlations of nuclear size with cell size in specific cell types of eukaryotic species.

### Nuclear Size Correlates Weakly With Erythrocytic Size Within Species

**FIGURE 2:**
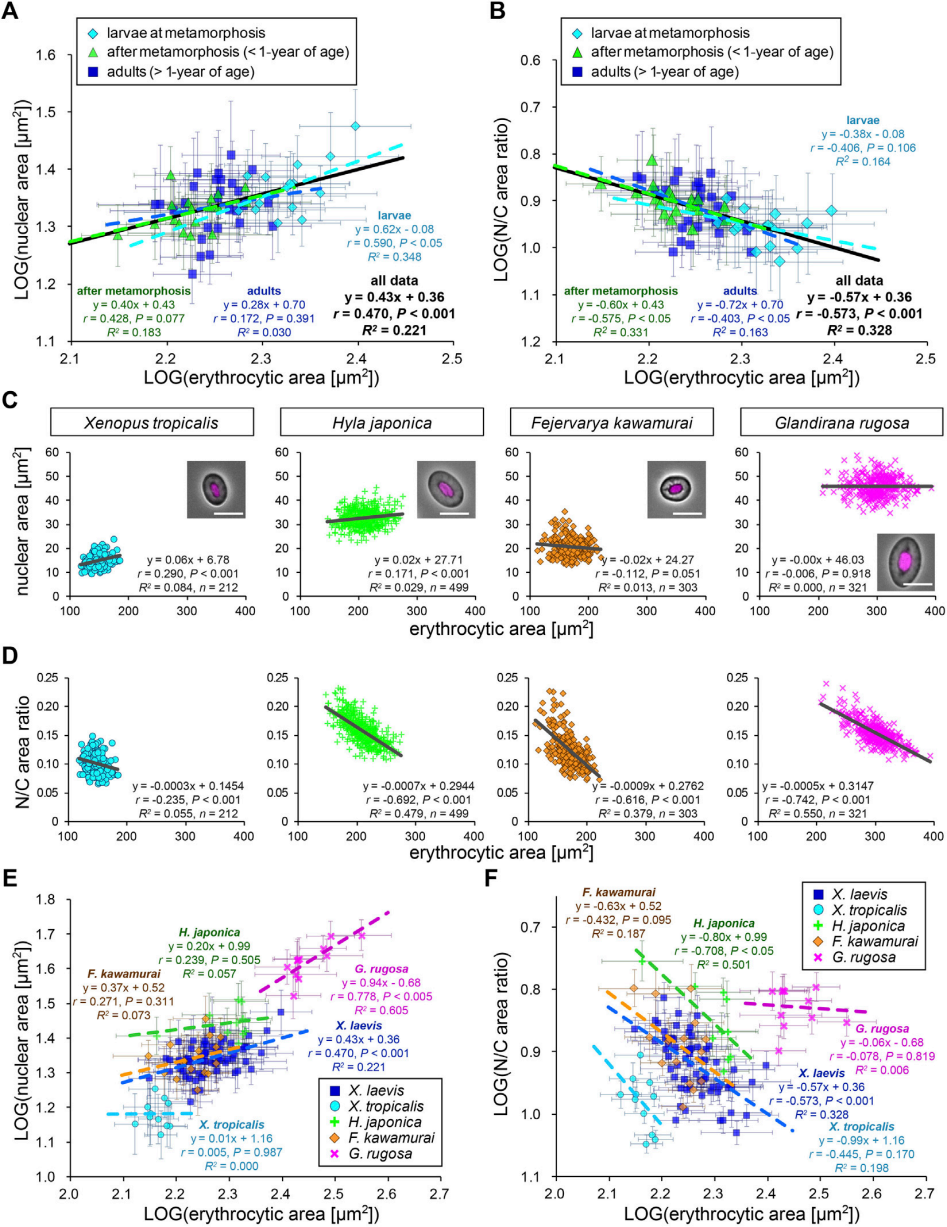
Comparison of nuclear cross-sectional areas with erythrocytic areas within each frog species. **(A)** Calculated mean nuclear cross-sectional areas were plotted against mean erythrocytic cross-sectional area in a log-log plot. Each symbol corresponds to the mean data (±SD) from each *X. laevis* individual at the stages of larva during metamorphosis (light blue, *n* = 17), young adult after metamorphosis (green, *n* = 18), or fully grown adult (blue, *n* = 27). The equation for the regression line, Pearson’s correlation (*r*), *P* values, and the coefficient of determination (*R* ^*2*^) have been indicated at each developmental stage (colored lines) and for the overall data (black). **(B)** The calculated mean value of the N/C area ratios were plotted against the mean erythrocytic area in a log-log plot. **(C)** The measured nuclear cross-sectional areas were plotted against the erythrocytic cross-sectional areas of adult frogs *X. tropicalis* (light blue), *H. japonica* (light green), *F. kawamurai* (orange), and *G. rugosa* (pink). We could not judge the exact age of these frogs, which were caught in a rice field. Each symbol corresponds to the data from each erythrocyte. A merged image of an erythrocyte with Hoechst 33342 (magenta) staining and phase contrast is shown in each graph. Scale bar = 10 μm. The equation for the regression line, sample number (*n*), *r*, *P* values, and *R* ^*2*^ have been indicated in each plot. **(D)** The calculated mean values of N/C area ratios were plotted against the measured erythrocytic areas. **(E)** The calculated mean nuclear cross-sectional areas were plotted against the mean erythrocytic cross-sectional area in a log-log plot. Each symbol corresponds to the mean data (±SD) from each frog and each species. The equation for the regression line, *r*, *P* values, and *R* ^*2*^ have been indicated in each species. *X. laevis*: *n* = 62; *X. tropicalis*: *n* = 11; *H. japonica*: *n* = 10; *F. kawamurai*: *n* = 16; *G. rugosa*: *n* = 11. **(F)** The calculated mean value of N/C area ratios have been plotted against the mean erythrocytic cross-sectional areas from each frog and each species in a log-log plot.

Next, to analyze the scaling of the nuclear size to the erythrocytic area within *X. laevis* species, we calculated the mean values in each frog and plotted the mean nuclear areas against mean erythrocytic areas in a log-log plot (Figure 2A). In this plot, the dataset showed a statistically significant (*p* < 0.001) correlation between the nuclear and erythrocytic areas. From a correlation analysis estimated as *(nuclear area) = A × (erythrocytic area)* ^*B*^, we detected a “hypoallometric” relationship, where the scaling exponent *B* was less than 1 (*B* = 0.43, *r* = 0.47, *p* < 0.001, the regression line shown in black in Figure 2A), suggesting that the relative nuclear area against erythrocytic area decreases when erythrocytic area increases. In line with this, when we calculated mean N/C area ratios in each frog and plotted them against mean erythrocytic areas, the N/C area ratio correlated negatively with the erythrocytic area in the dataset including all developmental data (*B* = −0.57, *r* = −0.57, *p* < 0.001, the regression line shown in black in Figure 2B). It should be noted that a statistically significant correlation between nuclear area and erythrocytic area was also obtained when the dataset was plotted on a normal graph with linear regression (*r* = 0.48, *p* < 0.001; Supplementary Figure S2C). The analysis of the correlation between the nuclear areas and cell areas at each developmental stage showed that the scaling exponent and strength of the correlation obtained from the dataset of larvae during morphogenesis (*B* = 0.62, *r* = 0.59, *p* < 0.05) were higher than those in fully grown adults (*B* = 0.28, *r* = 0.17, *p* = 0.39) and young adults (*B* = 0.40, *r* = 0.43, *p* = 0.08). Since the measured range of erythrocytic areas in the larval samples was wider than that in adults, the correlation of the nuclear areas with the erythrocytic areas might be more apparent in the larval samples. Although the size scaling feature of the nucleus changes during development, the intra-species correlation of the nuclear size with the erythrocytic size could be detected within the dataset from a single *X. laevis*, including intrinsic size variations during development.

To examine the consistency of the detected nuclear size scaling features in other frog species, we measured erythrocytes of other clawed frog species, *X. tropicalis*, and regional frog species *H. japonica*, *G. rugosa*, and *F. kawamurai*. The measured erythrocytic areas in *G. rugosa* were substantially larger than those in *X. laevis*, whereas those in *X. tropicalis* and *F. kawamurai* were smaller (Figure 2C). In each dataset obtained from the four frogs, the measured nuclear areas correlated very weakly (*r* < 0.4) with the erythrocytic areas in some samples of four frogs [*X. tropicalis*: *r* (±SD) = 0.23 ± 0.10; *H. japonica*: 0.16 ± 0.18; *G. rugosa*: 0.14 ± 0.10; *F. kawamurai*: 0.01 ± 0.10], similar to that in *X. laevis* erythrocytes (Figure 2C; Supplementary Table S1). Although the significant correlations (*p* < 0.05) were detected in some samples, the calculated slope values of the regression lines were very small in all frog samples [mean slope (±SD) = 0.05 ± 0.02 in *X. tropicalis*; 0.09 ± 0.19 in *H. japonica*; −0.01 ± 0.29 in *F. kawamurai*; 0.02 ± 0.02 in *G. rugosa*]. This result suggested that the measured nuclear area did not change significantly when the erythrocytic area increased in all frog species. In line with this, the calculated N/C area ratios showed a negative correlation with the erythrocytic area in every species, excluding samples of *X. tropicalis*, which showed a narrower erythrocytic size variation (Figure 2D; Supplementary Table S1). Furthermore, when we compared the measured nuclear areas with the calculated contour length or shape index, the nuclear areas still showed very weak correlations with the parameters for each erythrocytic shape (Supplementary Figure S3A). When we analyzed the intra-species correlation in a log-log plot using the mean values obtained from individual frogs, we observed a correlation between the nuclear areas and erythrocytic area in *G. rugosa* (*r* = 0.78, *p* < 0.005; Figure 2E). However, no statistically significant correlation was obtained in other species (*X. tropicalis: r* = 0.01, *p* = 0.99; *F. kawamurai*: *r* = 0.27, *p* = 0.31; *H. japonica*: *r* = 0.24, *p* = 0.51; Figure 2E). The calculated scaling exponent *B* of the nuclear areas to the erythrocytic areas revealed hypoallometry, with *B* < 1 in every species, although the values were distinct in each species. Furthermore, the N/C area ratio showed a negative correlation with the erythrocytic area in most species (Figure 2F), supporting the observed hypoallometric relationship between the nuclear area and the erythrocytic area. The dataset for *G. rugosa* showed no correlation between N/C area ratio and erythrocyte area (*r* = -0.08, *p* = 0.82), which is thought to be caused by the apparent correlation between the nuclear area and erythrocyte area. Overall, the data suggest that although the quantitative features of nuclear size scaling are different in each species, the lower correlation of nuclear size with erythrocytic size within individuals and the hypoallometric scaling within species might be conserved across frog species.

### Erythrocytic Size and Nuclear Size Correlate Differently With Genomic Content Among Species

**FIGURE 3:**
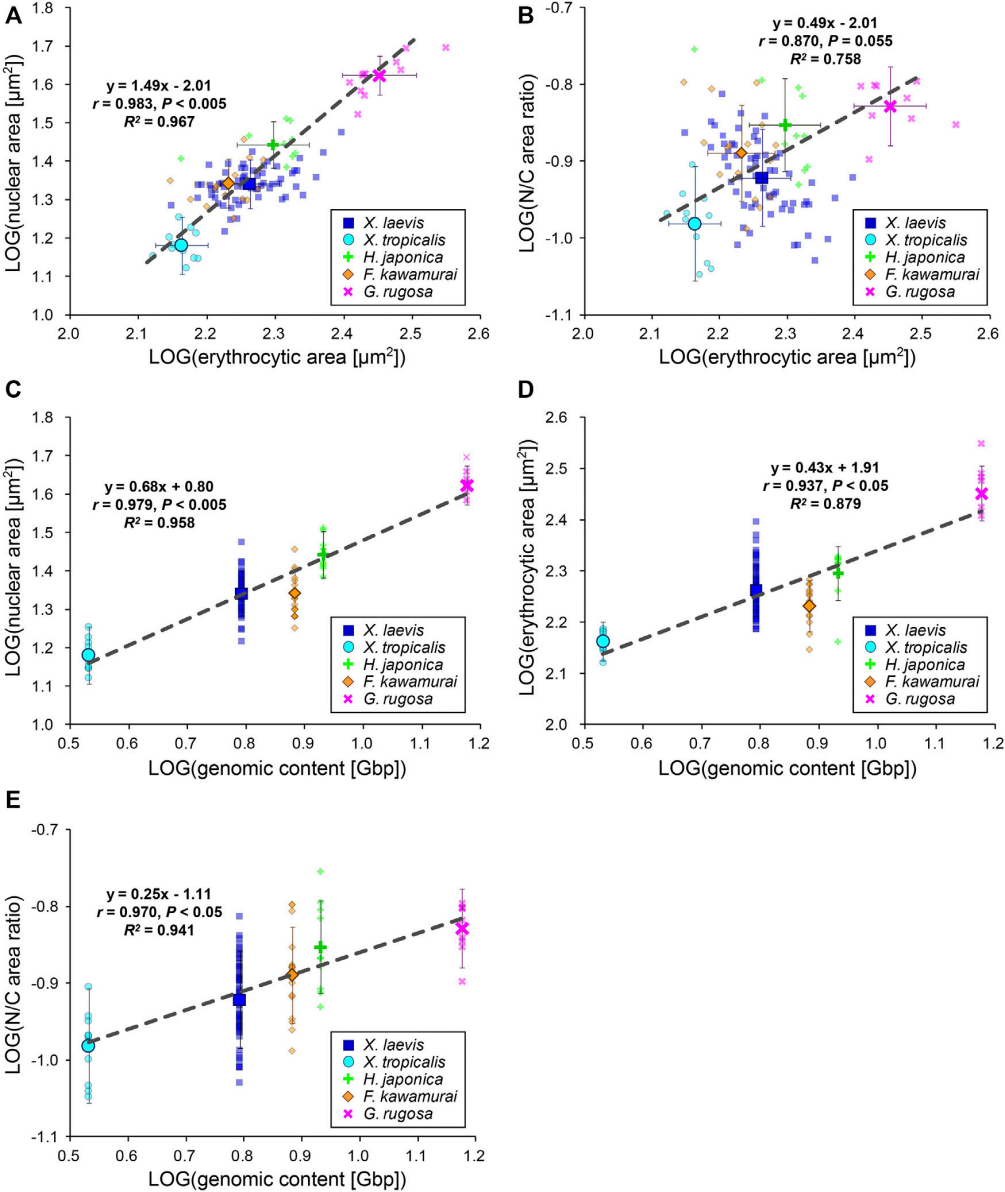
Inter-species relationship among nuclear area, erythrocytic area, and genomic content in five frog species. **(A)** Calculated mean nuclear cross-sectional areas and **(B)** calculated mean N/C area ratios of each species were plotted against the mean erythrocytic cross-sectional areas in a log-log plot. **(C)** Calculated mean nuclear cross-sectional areas, **(D)** mean erythrocytic cross-sectional areas, and **(E)** mean N/C area ratios from each species were plotted against the genomic content in a log-log plot. The mean data from each frog (small symbols) and mean data from each species (big symbols; ±SD) have been shown, along with the equation for the regression line, Pearson’s correlation (*r*), *P* values, and coefficient of determination (*R* ^*2*^) in each plot. The regression lines were calculated using mean data from each species. *X. laevis*: *n* = 62; *X. tropicalis*: *n* = 11; *H. japonica*: *n* = 10; *F. kawamurai*: *n* = 16; *G. rugosa*: *n* = 11.

To evaluate the inter-species size scaling relationship, we calculated the mean values for each species and plotted the mean nuclear areas against the mean erythrocytic areas in a log-log plot (Figure 3A). In contrast to the low scaling exponents in the intra-species datasets, the inter-species scaling exponent *B* was more than one among the five different species (*B* = 1.49, *r* = 0.98, *p* < 0.005), indicating a hyperallometric relationship in which the relative nuclear area *versus* the erythrocytic area increases when the erythrocytic area increases. In line with this, the calculated N/C area ratio revealed a positive correlation with erythrocytic area (Figure 3B; *B* = 0.49, *r* = 0.87, *p* = 0.06). Next, to consider the effects of genomic content, which is intrinsically different in each species, we compared the mean nuclear areas of each species with the genomic content within the nucleus (Figure 3C). In the plots, the nuclear area correlated well with the genomic content (*r* = 0.98, *p* < 0.005). In accordance with the well-known relationship between erythrocytic size and genomic content, our measured erythrocytic area also correlated with the genomic content (Figure 3D; *r* = 0.94, *p* < 0.05). Although both the nuclear and erythrocytic areas qualitatively correlated with the genomic content, the calculated scaling exponent of the erythrocytic area to the genomic content (*B* = 0.43) was substantially smaller than that of the nuclear area to the genomic content (*B* = 0.68). This difference in the scaling exponents indicates that the relative changes in the nuclear and erythrocytic areas are distinct when genomic content increases. This scaling feature was also supported by the positive correlation of the calculated N/C area ratio with the genomic content among the five frog species (Figure 3E; *B* = 0.25, *r* = 0.97, *p* < 0.05). It should be noted that statistically significant correlations between the nuclear area and erythrocytic area were also seen when the dataset was plotted on a normal graph using a linear regression (Supplementary Figure S3B). Overall, based on our inter-species comparisons with the genomic content, we found differences in the nuclear size scaling between intra- and inter-species and in the scaling exponents of the nuclear and erythrocytic sizes with the genomic content among the five limited frog species.

### Uniqueness of Size Scaling Features in Anura Erythrocyte Nuclei

**FIGURE 4:**
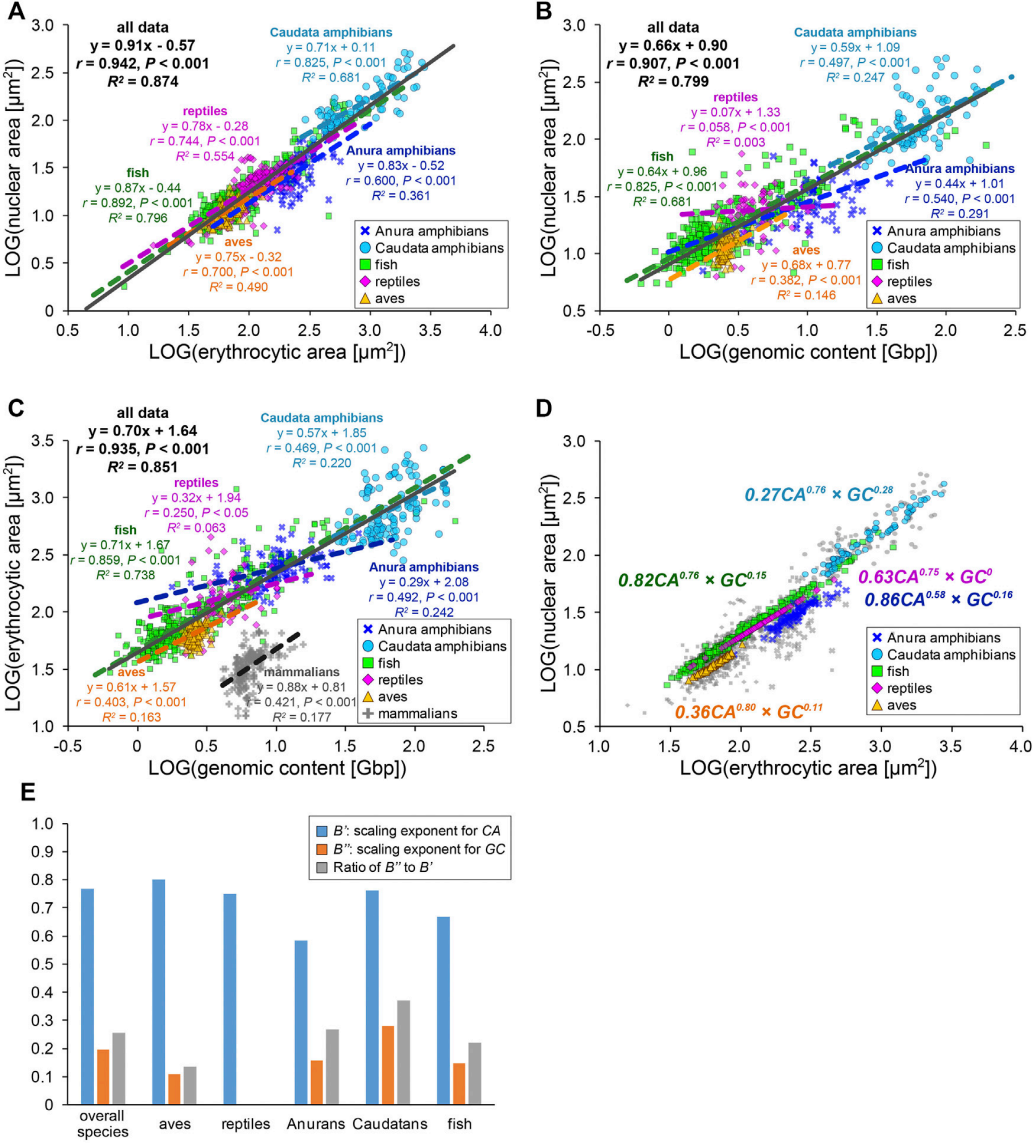
Inter-species relationship among nuclear area, erythrocytic area, and genomic content in different species classes. **(A)** Collected nuclear cross-sectional areas were plotted against erythrocyte cross-sectional areas in a log-log plot. The equations for the regression line, Pearson’s correlation (*r*), *P* values, and the coefficient of determination (*R* ^*2*^) have been indicated for datasets of each species class (colored) or all species together (gray). Data from Anura amphibians (blue, *n* = 93), Caudata amphibians (light blue, *n* = 88), fish (green, *n* = 354), reptiles (purple, *n* = 157), and aves (yellow, *n* = 270) have been shown. **(B)** Collected nuclear and **(C)** erythrocytic cross-sectional areas have been plotted against the genomic contents in a log-log plot. The dataset of enucleated erythrocytes from mammalian species have also been plotted (gray). Anura amphibians: *n* = 71 **(B)**, *n* = 105 **(C)**; Caudata amphibians: *n* = 86 **(B)**, *n* = 122 **(C)**; fish: *n* = 336 **(B)**, *n* = 383 **(C)**; reptiles: *n* = 62 **(B)**, *n* = 64 **(C)**; aves: *n* = 124 **(B)**, *n* = 186 **(C)**; and mammalians: *n* = 204 **(C)**. **(D)** Nuclear cross-sectional areas estimated from the equation with two scaling exponents, for erythrocytic cross-sectional area *CA* and genomic content *GC*, have been shown using colored symbols. Gray symbols are identical to the collected dataset shown in **(A)**. The scaling exponents estimated using the equations for each species class have been shown in the plot. **(E)** Estimated values of the scaling exponents for *CA* (blue), *GC* (orange), and ratios of the scaling exponent for *CA* to that for *GC* (gray) in each dataset of species class and that for all species together.

To extensively understand whether our observed size scaling features are conserved among other amphibians and species in the other classes, we revisited the prominent open databases of nuclear size, erythrocytic size, and genomic size collected by Ryan T. Gregory across a wide range of species (Animal Genome Size Database, Cell Size Database). Furthermore, we combined our original measurement dataset of five frog species and our collected data from published papers into Gregory’s datasets. To analyze the effects of either genomic content or cell size on the determination of the nuclear size separately, we excluded the data in which one parameter was predicted from the other, unlike previous comparisons (Malerba and Marshall, 2021; Gillooly et al., 2015). Using our compiled dataset, we analyzed relationships among the three scaling parameters in each species class: Anura and Caudata amphibians, aves, fish, and reptiles (Figure 4, Supplementary Table S2). Although our compiled dataset should have variations in the size parameters, due to potential differences in data sampling, the qualitative size relationship between two parameters appeared to be conserved across the species class. In the amphibian dataset from Caudatans or Anurans, the scaling exponents of the nuclear areas to the erythrocytic areas revealed a hypoallometry with *B* < 1 (*B* = 0.71, *r* = 0.82, *p* < 0.001 in Caudatans; *B* = 0.83, *r* = 0.60, *p* < 0.001 in Anurans; Figure 4A). When calculating *B* values from the dataset of other species classes, the hypoallometric scaling of the nuclear areas to the erythrocytic areas was conserved in the fish, reptiles, or aves class, with *B* values maintained within the range of 0.75–0.87 (Figure 4A). Although the calculated *B* values revealed variations among species classes, the hypoallometric scaling of the nuclear size to the erythrocytic size was well conserved among all species classes (*B* = 0.91, *r* = 0.94, *p* < 0.001; black line in Figure 4A). This hypoallometry in erythrocytes is consistent with the previous calculations in inter-species datasets, including non-biased cell types (Hara, 2020; Malerba and Marshall, 2021).

Next, to analyze the contribution of the genomic content among species, the nuclear areas were plotted against the genomic contents in each species class (Figure 4B). In this plot, the datasets of fish or amphibians revealed a positive correlation of the nuclear areas to the genomic content (*r* = 0.50–0.83, *p* < 0.001), which is consistent with our original measurement from five frogs. On the contrary, in the dataset of reptiles or aves, the nuclear areas revealed a very weak correlation with the genomic content (*r* < 0.40, *p* < 0.001; Figure 4B). Furthermore, the ratios of the explained variation from the regression line is larger in fish or amphibians (*R* ^*2*^ = 0.25–0.68) than those in reptiles or aves (*R* ^*2*^ = 0.00–0.15). In addition to the differences in the strength of the scaling, the calculated scaling exponent in Anurans (*B* = 0.44) was lower than that in fish or Caudatans (*B* = 0.64, 0.59; Figure 4B). These calculated values of *r* (0.54) and *B* in Anurans varies with those observed in our original measurement of five frog species (*r* = 0.98, *B* = 0.68; Figure 3C), suggesting that the scaling features and underlying mechanisms might vary even within each species class and might change independently during evolution from fish into each class. We further plotted the erythrocytic areas against the genomic contents in the datasets of classes with nucleated and enucleated erythrocytes in mammalian species (Figure 4C). Positive correlations with a hypoallometric scaling relationship were detected in the dataset of nucleated erythrocytes over species classes (*B* = 0.70, *r* = 0.94, *p* < 0.001) and most datasets of individual classes (Figure 4C). Interestingly, the strength of the correlation and the values of the scaling exponent were different for each class. For instance, the fish dataset revealed a clear correlation of the erythrocytic area with the genomic content (*r* = 0.86, *p* < 0.001), while the correlations in other classes, including mammalians with enucleated erythrocytes, were not apparent (*r* = 0.25–0.47, *p* < 0.001). It should be noted that when the datasets were plotted on a normal graph using a linear regression, the correlations among the three parameters were conserved (Supplementary Figure S4). However, the correlation coefficients (*r*) and coefficient of determination (*R* ^*2*^) in some samples were reduced when analyzing the normal plot that used linear regression (e.g., *r* = 0.54, *R* ^*2*^ = 0.29 in scaling analysis of nuclear area with genomic content among Anura amphibians; *r* = 0.36, *R* ^*2*^ = 0.13 in the normal plot). Therefore, we used scaling analyses to better understand these scaling features.

We hypothesize that such species- and/or class-specific scaling features are derived from the differences in the contribution of genomic content and erythrocytic size in the determination of the nuclear size. To distinguish the two contributions to nuclear size determination, the datasets were fitted to an equation combining two power-law parameters, *[nuclear area (NA)] = A’ × [erythrocytic area (CA)]* ^*B’*^ *× [genomic content (GC)]* ^*B’’*^. Although the mutual relationship among *NA, CA,* and *GC* might be complex, in the present study, we simply assumed the equation to be a product of powered *GC* and powered *CA*. Based on the *CA*, *GC*, and *NA* values, the constant *A’* and scaling exponents *B’* and *B’’* were estimated using the least-squares method in each class (Figures 4D,E). Because the estimated *NA* values for each group were used, the fitness of the estimated *NA* to the real collected *NA* was better than when the estimated *A* and *B* values from the equations with single-powered parameters, such as *(NA) = A × (CA)* ^*B*^ or *(NA) = A × (GC)* ^*B*^ were used (Supplementary Figure S5). The estimated scaling exponents *B’* for *CA* in the equation with multiplied *GC* and *CA* (B’ = 0.58–0.80, Figures 4D,E) decreased from the scaling exponents *B* estimated using the single powered *CA* in each class (*B* = 0.71–0.87, Figure 4A). Furthermore, the estimated scaling exponents *B’’* for *GC* in the equation (*B’’* = 0–0.28, Figures 4D,E) were far smaller than *B* estimated using the single powered *GC* (*B* = 0.07–0.68, Figure 4B). In extreme cases in reptiles, even if both *GC* and *CA* are considered for the parameter estimation, the fitness is no longer improved (Figures 4D,E), suggesting that the contribution of the genomic contents in determination of the nuclear size might be very small in reptile erythrocytes. To clarify the difference between the two scaling exponents for *CA* and *GC*, we calculated the ratio of the two values of the scaling exponents *B’’* to *B’* (Figure 4E). As a result, the ratios were substantially higher in amphibians than those in aves and reptiles, suggesting that the contribution of the genomic content in determination of the erythrocyte nuclear size might have been switched during evolution from aquatic organisms to terrestrial organisms. Collectively, erythrocytes have a unique inter-species size scaling feature because of the low correlation of the genomic content, which reveals species-specificity in the degree of the contribution of the genomic content in the determination of nuclear size.

**FIGURE 5:**
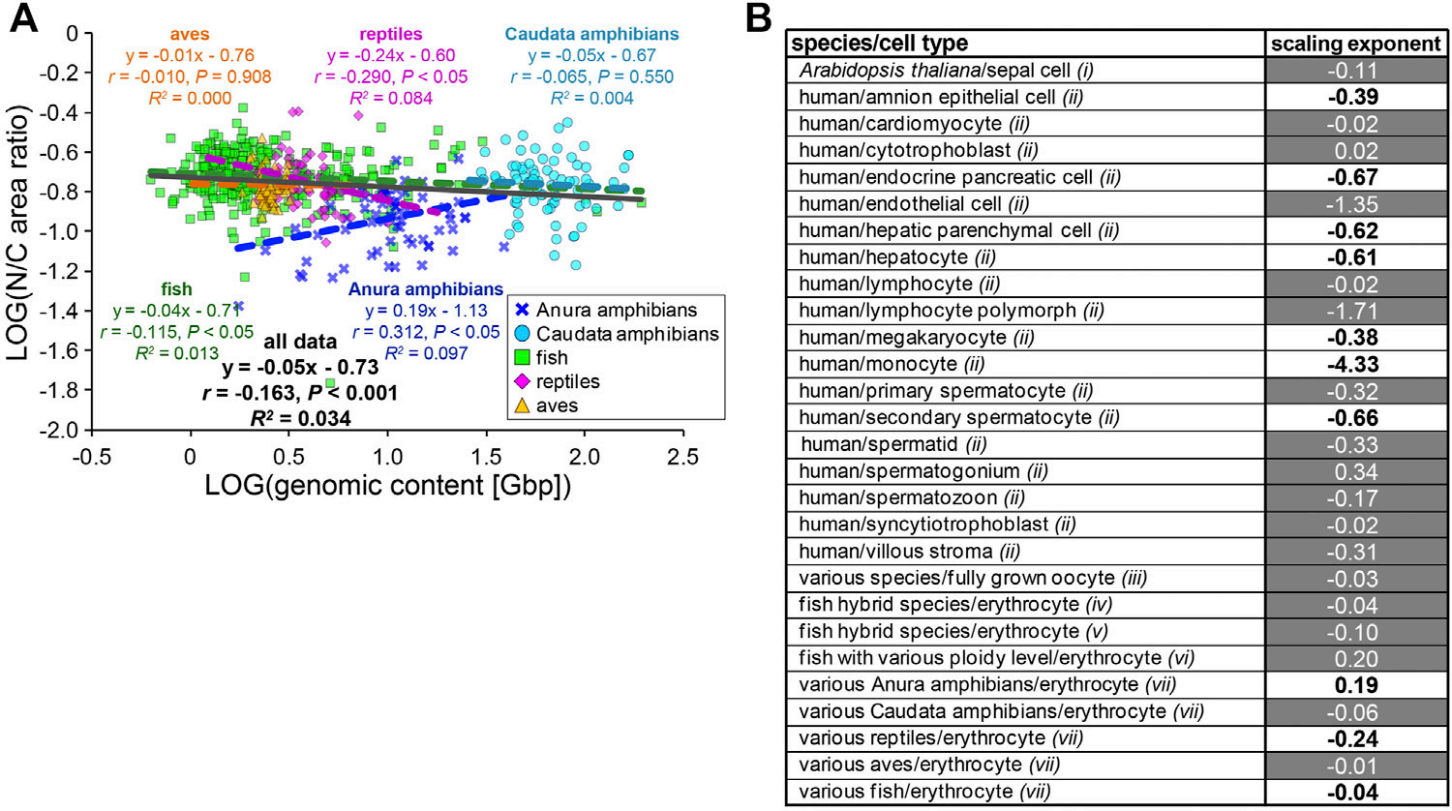
Comparison of N/C area ratio with genomic content. **(A)** Collected N/C area ratios were plotted against the genomic contents in a log-log plot. Anura amphibians: *n* = 71; Caudata amphibians: *n* = 80; fish: *n* = 317; reptiles: *n* = 62; aves: *n* = 124. The equation for the regression line, Pearson’s correlation (*r*), *P* values, and the coefficient of determination (*R* ^*2*^) have been indicated in the plot. **(B)** Calculated scaling exponents of the N/C area ratio to the genomic content in each dataset from single cell-types with different ploidy levels, from single species or various species. Datasets were collected from published papers; *i*: measured from graphs in Robinson et al., 2018; *ii*: listed in Gillooly et al., 2015; *iii*: listed in Hara, 2020; *iv*: measured from images in Lu et al., 2009; *v*: measured from images in Hu et al., 2016; *vi*: listed in Gao et al., 2007; *vii*: this study. Black and white values in the column represent samples with the statistically significant (*p* < 0.05) correlations (*r*) and no significant correlation, respectively.

The observed variations in the contributions of the genomic content and erythrocytic size in the determination of nuclear size are expected to change the relationship between N/C area ratio with *GC* in each species. When the calculated N/C area ratio versus the *GC* was plotted, the N/C area ratio correlated very weakly with the *GC* in each species and for the entire dataset across species classes (*r* < 0.4; Figure 5A). This might result from our estimated relatively low contribution of the *GC* in determining the *NA*, as compared to *CA*. To ascertain whether this scaling feature of the N/C area ratio is specific for erythrocytes, we calculated the scaling exponents of the N/C area ratio to the *GC* using datasets including variation of the genomic contents and nuclear size from published studies with data of various cell types among species or single cell types with different ploidy levels from a single organism (Figure 5B). As a result, the most frequently calculated values of *B* were approximately 0, with no significant correlation (*p* > 0.05) in the datasets of erythrocytes and some other cell types, as observed in our erythrocyte datasets (Figure 5B). The *B* values in most human cell types were substantially smaller than 0 and had significant correlations (Figure 5B), indicating that the relative nuclear size *versus* cell size decreases when the genomic size increases. In general, in eukaryotic cells other than erythrocytes, an increase in genomic content might contribute more to the weakening increase in nuclear size. Therefore, lower correlations of the genomic content to the nuclear size and N/C area ratio could be specific features that could help characterize erythrocyte-specific size scaling and functions.

### Specificity of Nuclear Structures in Frog Erythrocytes

**FIGURE 6:**
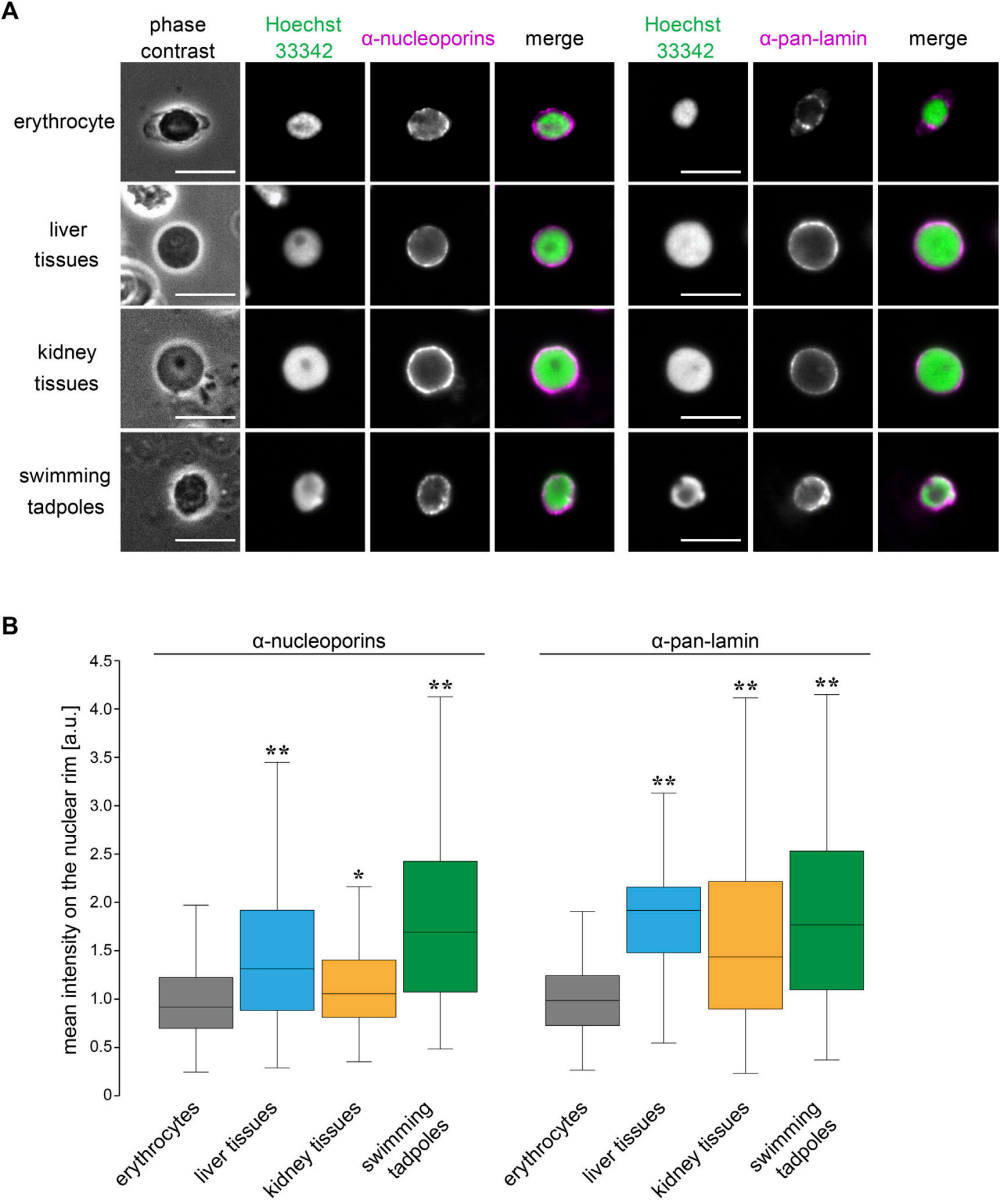
Structural properties of nuclear membrane in *X. laevis* erythrocytes.(A) Immunostaining using anti-nucleoporins (left; magenta) or anti-pan-lamin (right; magenta) antibodies, with DNA staining using Hoechst 33342 (green), in erythrocytes, isolated nuclei from liver, kidney cells, and swimming tadpoles of *X. laevis*. Merged images of each immunostaining signal with DNA signals have been shown. Scale bar = 10 μm. (B) Mean intensity of the immunostaining signals for anti-nucleoporins or anti-pan-lamin antibodies was represented in a box plot. The measured intensities from erythrocytes and other cells were normalized to the mean intensity of erythrocytes. Samples number (n) from erythrocytes: 71 (nucleoporins), 105 (lamins); liver cells: 86 (nucleoporins), 122 (lamins); kidney cells: 86 (nucleoporins), 122 (lamins); swimming tadpoles: 86 (nucleoporins), 122 (lamins). The statistical difference between data from erythrocytes and other tissues and embryos was analyzed using the Wilcoxon test (**p < 0.005 and *p < 0.05).

To explore which nuclear features contribute to the size scaling specificity in erythrocytes, we analyzed the nuclear structure in *X. laevis* erythrocytes by visualizing the nuclear membrane constituents. Immunostaining using anti-nucleoporin antibodies that recognize multiple NPC components and anti-pan lamin antibodies that recognize almost all lamin isoforms revealed nuclear pores and lamins in the peripheral region of the DNA (Figure 6A). The distribution of the nuclear pores and lamins took the form of a dot-like pattern on the rim of the erythrocyte nucleus. In contrast, these nuclear membrane constituents were located continuously on the nuclear rim without large gaps in the nuclei isolated from the liver and kidney tissues as well as tadpole embryos (Figure 6A). Furthermore, the mean intensities of both nucleoporin- and lamin-positive signals were significantly lower in erythrocytes than those in other tissues and embryos, although the intensities differed among nuclei from tissues and embryos (Figure 6B). The detected reduction in the mean signal intensity was caused by both a reduction in the quantity of nuclear pores and lamins and changes in the distribution on the nuclear rim. Overall, the observed reduction in the nuclear membrane constituents might be specific features of erythrocytes and involved in the control of erythrocyte-specific nuclear size scaling with genomic content.

## Discussion

The nuclear size scaling in erythrocytes varies between intra- and inter-species datasets. In the intra-species datasets that showed intrinsic size variations in erythrocytes from individual frogs, the nuclear size correlated very weakly with erythrocytic size (Figure 1). Upon increasing the erythrocytic size ranges, by collecting erythrocytes from different individuals at various developmental stages, the nuclear size showed a weak hypoallometric relationship with erythrocytic size (Figure 2). On the contrary, upon analyzing the scaling relationship over species with much larger size variations, the nuclear size showed an almost proportional relationship and a stronger correlation with the erythrocytic size (Figures 3, 4). Thus, although the observed much weaker correlations in the intra-species datasets might be caused by the limited ranges of erythrocytic size variations, the weak strength of the correlation and smaller scaling exponent of the nuclear area to the erythrocytic area are clearly distinct from the stronger correlation and almost proportional relationship found in the datasets with intrinsic size variation of certain cell types, such as culture cells in human (Viana et al., 2020), shoot meristems in flowering plants (D’Ario et al., 2021), yeasts (Jorgensen et al., 2007; Neumann and Nurse, 2007), and green alga (Malerba and Marshall, 2021). Regarding the mechanisms underlying the control of nuclear size based on the cytoplasm, our observations showed that nuclear membrane constituents, including NPCs, which control the nuclear import of proteins and lamins (Levy and Heald, 2010), are riddled on nuclear membranes (Figure 6). Thus, the known cytoplasm-based mechanisms for controlling the nuclear size are expected to be less effective in erythrocytes. Nonetheless, a superficial correlation of the nuclear size to the erythrocytic size was observed in inter-species datasets. To explain this scaling relationship, we assumed that the scaling of the nuclear size to the erythrocytic size is predefined before the development of erythrocytes from erythroids, which is referred to as erythropoiesis, accompanied by a reduction in erythroid cytoplasm, rather than being controlled in the terminally differentiated erythrocytes. This assumption also supports the resulted correlation between erythrocytic size and genomic content in mammalian enucleated erythrocytes without DNA inside. Thus, in future studies, it would be worth investigating the nuclear size scaling relationship and the underlying mechanisms during erythropoiesis, to comprehensively understand how nuclear size scaling is achieved in erythrocytes.

The erythrocyte nuclear size also correlates with the genomic content in our observed frog species and collected amphibian datasets (Figures 3, 4). In the scaling analysis using two parameters, erythrocytic area and genomic content, the scaling exponent of the genomic content to the nuclear area is commonly smaller than that to the erythrocytic area in each species class and all species classes together (Figure 4D). The obtained difference between the two scaling exponents suggested that the contribution of erythrocytic size for nuclear size determination is higher than that of genomic content. This lower contribution of genomic content might be caused by the putative lower contact frequency of chromatin to the nuclear membrane in erythrocytes. Upon microscopic observation of the frog erythrocytes (Figure 6), we found a reduction in the quantity and riddled distribution of nuclear structural constituents such as NPCs and lamins. This reduction in nuclear membrane constituents was also observed during erythropoiesis in vertebrates. The quantity of nuclear membrane constituents and the nuclear size are reduced upon formation of multiple gaps of the lamina and NPCs on the nuclear membrane (Maul et al., 1980; Makatsori et al., 2004; Zhao et al., 2016; Zhen et al., 2020). Considering that the inner nuclear envelope proteins are in contact with chromatin (van Steensel and Belmont, 2017), a lower quantity of envelope proteins should weaken the interaction of chromatin with the nuclear envelope. Indeed, this connection helps in the transmission of forces from the chromatin to the nuclear membrane (Schreiner et al., 2015; Stephens et al., 2017). During the nuclear expansion phase, the repulsion force of chromatin helps in the expansion of the nuclear membrane from inside the nucleus (Heijo et al., 2020). Furthermore, if the nuclear size expands excessively through a large supply of lipid membranes or the chromatin becomes excessively compacted by hyper-condensation, the chromatin pulls the nuclear membrane, resulting in stalling or interruption of nuclear expansion (Chan et al., 2017; Shimamoto et al., 2017; Stephens et al., 2018; Heijo et al., 2020). This experimental evidence suggests that the strength of the contribution of chromatin to nuclear size determination should be reduced in the riddled nuclei of frog erythrocytes. Furthermore, because the chromatin within erythrocyte nuclei is highly condensed (Ji et al., 2011; Zhao et al., 2016), the degree to which the genomic contents contribute to size or expansion of the nucleus is expected to be small.

Considering the observed erythrocyte-specific nuclear size scaling and nuclear structures, we hypothesized that there is a differential balance in the two scaling pathways related to genomic content and cell size in each cell type and species. In the datasets of erythrocytes, when estimating the two scaling exponents for erythrocytic area and genomic content to the nuclear area in our scaling analysis, the values for each scaling exponent were different among species classes (Figure 4). In particular, in the datasets collected from the Anura and Caudata amphibians, the ratios of the scaling exponent of genomic content to that of the erythrocytic area were higher than those in other classes (Figure 4E). This difference could be explained in terms of the differences in the structural properties of the nuclear membrane. In avian erythrocytes, lamin proteins are known to be located on the nuclear membrane without gaps, although the lamin-B receptor proteins are distributed in a dot-like pattern on the nuclear membrane (Makatsori et al., 2004), which differs from the observed distribution of lamins in frogs. In mice erythroid, just before enucleation into reticulocytes, the nuclear envelope with lamin B remains intact, without any gaps (Krauss et al., 2005). Amino acid sequences of nuclear envelope constituents such as lamins and NPCs and the quantitative amount of lamin isoforms are also known to be different among eukaryotic species and cell types (Mans et al., 2004; Swift et al., 2013). In addition to the structural properties, the difference in the nuclear shape may also affect the scaling exponent of the genomic content. In erythrocytes of sturgeon species, the ratio of nuclear surface area to nuclear volume, which corresponds to the nuclear shape, is different among erythrocytes with different ploidy levels (Bytyutskyy et al., 2014). In addition, the higher-order chromatin structures within erythrocyte nuclei, such as topologically associated domains and long-range interactions of chromatin intra-chromosomes, vary to some extent among species classes (Ryzhkova et al., 2021). These evolutionary differences, in not only the structural properties of the nuclear envelope but also the physical and epigenetic status of chromatin, presumably change the contribution of genomic content to nuclear size determination in each species. The putative changes in the contribution of genomic content should result in a change in the N/C size ratio, depending on the genomic content. From our scaling analysis, the scaling exponent *B* of the N/C area ratio to the genomic content varied in each dataset from other cell types with various ploidy levels (Figure 5B). In the datasets of oocytes among various species (Hara, 2020), the scaling exponent *B* of the N/C area ratio was approximately 0, with no significant correlation, which was similar to that in erythrocytes (Figure 5B). The contribution of cytoplasm-based mechanisms in determining the nuclear size *via* nuclear protein import and membrane supply is much stronger in the condition of huge cytoplasmic volume in the oocytes. In addition, the scaling exponents *B* of the N/C area ratio were also approximately 0 in datasets of a flowering plant with different ploidy levels (Robinson et al., 2018) and erythrocytes (Figure 5B). Under these conditions, the effective contribution of the chromatin may be reduced through less interaction of the chromatin with the plant-specific lamina composition, due to no expression of the canonical lamin genes and riddled distribution of the nuclear constituents, as described above. In contrast, in the datasets of various cell types with different ploidy levels from humans (Gillooly et al., 2015), the scaling exponent *B* of the N/C area ratio to the genomic content showed negative values with significant correlations (Figure 5B). Due to the nuclear lamina, which is especially developed in vertebrate species (Mans et al., 2004), more stable interaction of the chromatin with nuclear membrane could pull the nuclear membrane more inward, upon an increase in the genomic content. Indeed, highly compacted chromatin is a common feature of the terminal differentiation of other cell types, including lens epithelial cells, lymphocytes, and megakaryocytes, which generates a stronger pulling force on the nuclear membrane, resulting in weakening of the nuclear expansion. Collectively, we propose that the scaling properties of the N/C size ratio upon changing genomic content show a difference in the degree of chromatin and cytoplasmic contributions, to determine the nuclear size in each species and cell type.

During erythropoiesis, chromatin compaction is associated with the widespread downregulation of gene expression (Wong et al., 2011). Manipulation of epigenetic status, such as histone modification or conversion of nuclear membrane constituents, has been known to perturb chromatin condensation, enucleation events, and terminal differentiation in mouse erythropoiesis, leading to hematological diseases (Ji et al., 2011; Zhao et al., 2016). Therefore, our observed erythrocyte-specific scaling features are also expected to be important for physiological functions of erythrocytes. When the nuclear size is reduced during erythropoiesis in mammalian species, there is an increase in the hemoglobin concentration of the cytoplasm (Ji et al., 2011). Furthermore, erythrocytic size negatively correlates with hemoglobin concentration in humans (Di Caprio et al., 2015). These evidences suggest that erythrocytes control the amount of hemoglobin in the cytoplasm by manipulating the nuclear size, which excludes the functional cytoplasmic space. This assumption might also be applicable to increase hemoglobin levels in enucleated mammalian erythrocytes (Ji et al., 2011). Furthermore, our observed decrease in the scaling exponents of the genomic content to the nuclear size during the evolution from amphibian to avian and reptile classes (Figure 4E) allows us to anticipate the occurrence of future enucleation events during evolution. Indeed, in some salamander species, which have almost zero contribution of genomic content to nuclear size (Figure 4E), enucleation events occur during erythropoiesis (Villolobos et al., 1988; Mueller et al., 2008). Further systematic inter-species comparison of the quantitative features and underlying molecular mechanisms of nuclear size scaling during erythropoiesis are informative to elucidate why the nuclear size is controlled in erythrocytes and the transcriptionally silenced nucleus still exists in most vertebrate species from the viewpoint of size scaling.

## Data Availability

The original contributions presented in the study are included in the article/Supplementary Material, further inquiries can be directed to the corresponding author.
