# Supplementary material for: Specificity of Nuclear Size Scaling in Frog Erythrocytes
Source: Front Cell Dev Biol. 2022 May 18;10:857862. doi: 10.3389/fcell.2022.857862 (PMC9159806; doi:10.3389/fcell.2022.857862)
Supplement: Supplementary file 2 [file DataSheet1.pdf]

Supplementary Materials

Specificity of nuclear size scaling in frog erythrocytes

Tetsufuki Niide, Saki Asari, Kosuke Kawabata, Yuki Hara

Supplementary Figures

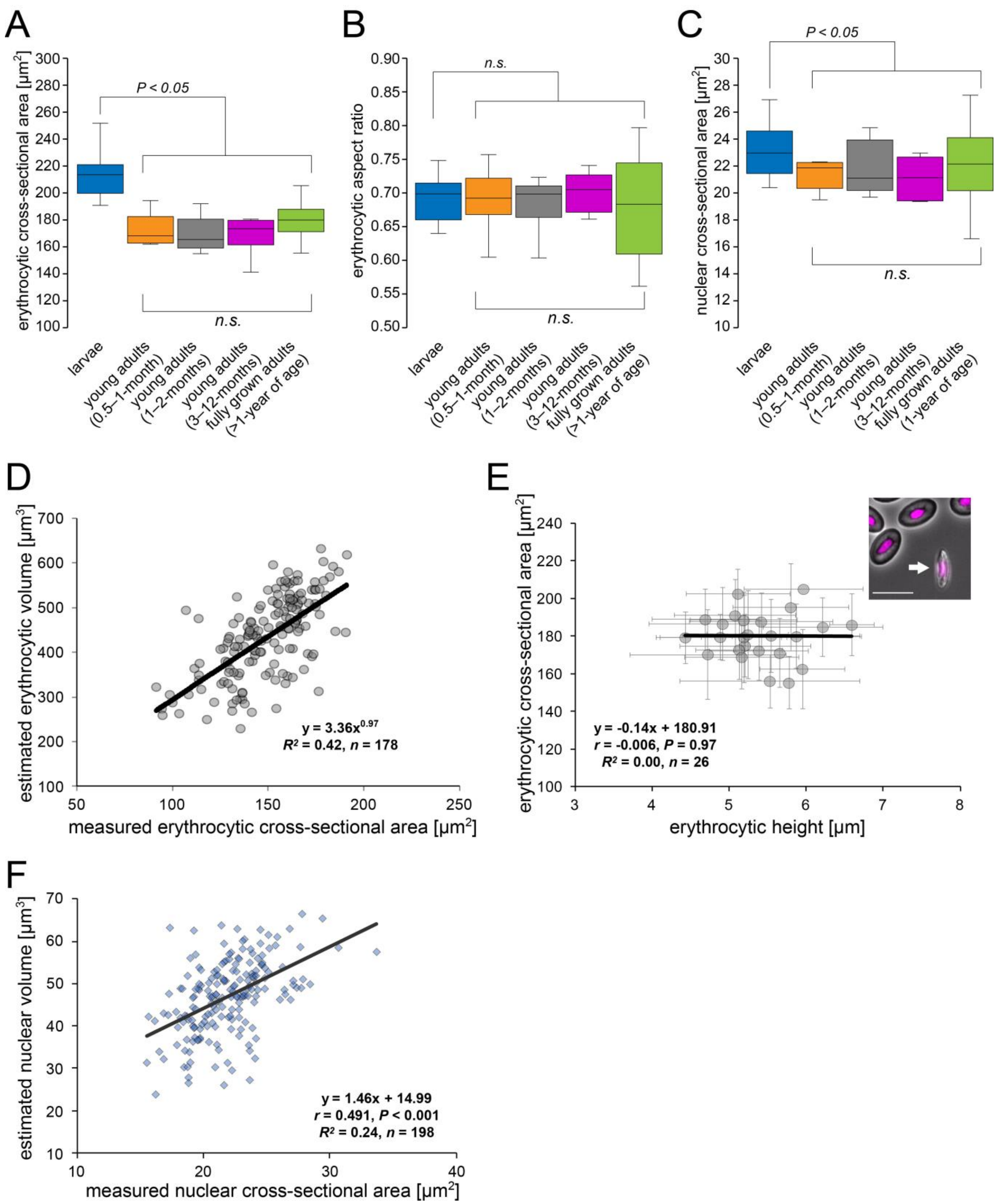

Supplementary Figure 1. (**A**) Mean measured erythrocytic cross-sectional area, (**B**) aspect ratio, or nuclear cross-sectional area (**C**) in each developmental stage was represented in a box plot. For cross-sectional areas (**A**) and (**C**), larva during metamorphosis, *n* = 17; young adults (2-weeks—1 month after metamorphosis), *n* = 6; young adults (1–2 months after metamorphosis), *n* = 6; young adults (3–12 months after metamorphosis), *n* = 6; fully grown adults (> 1-year of age), *n* = 27. For aspect

ratio (**B**), larva during metamorphosis, $n = 10$; young adults (2-weeks—1 month after metamorphosis), $n = 6$; young adults (1–2 months after metamorphosis), $n = 6$; young adults (3–12 months after metamorphosis), $n = 6$; fully grown adults (> 1-year of age), $n = 20$. The statistical differences between data among developmental stages were analyzed using a Wilcoxon Student's t-test ($P < 0.05$, *n.s.* = not significant difference). (**D**) Estimated erythrocytic volumes were plotted against the erythrocytic cross-sectional areas. Volumes were estimated using three-dimensional reconstruction. Cross-sectional areas were measured directly. Each symbol corresponds to data from each erythrocyte. The equation for the regression line with scaling exponent, sample number (n), and the coefficient of determination (R^2) have been indicated in the plot. (**E**) Calculated mean erythrocytic heights were plotted against the mean erythrocytic cross-sectional area from individual frogs. Each symbol corresponds to the mean data (±SD) from each frog. The equation for the regression line, n, Pearson's correlation (r), P values, and R^2 have been indicated in the plot. A merged image of *X. laevis* erythrocytes with Hoechst 33342 (magenta) staining and phase contrast is shown. A representative erythrocyte which we measured the height is indicted by an arrow. Scale bar = 20 µm. (**F**) Estimated nuclear volumes were plotted against the nuclear cross-sectional areas. Each symbol corresponds to data from each erythrocyte. Volumes were estimated using three-dimensional reconstruction. Cross-sectional areas were measured directly. The equation for the regression line, n, Pearson's correlation (r), P values, and R^2 have been indicated in the plot.

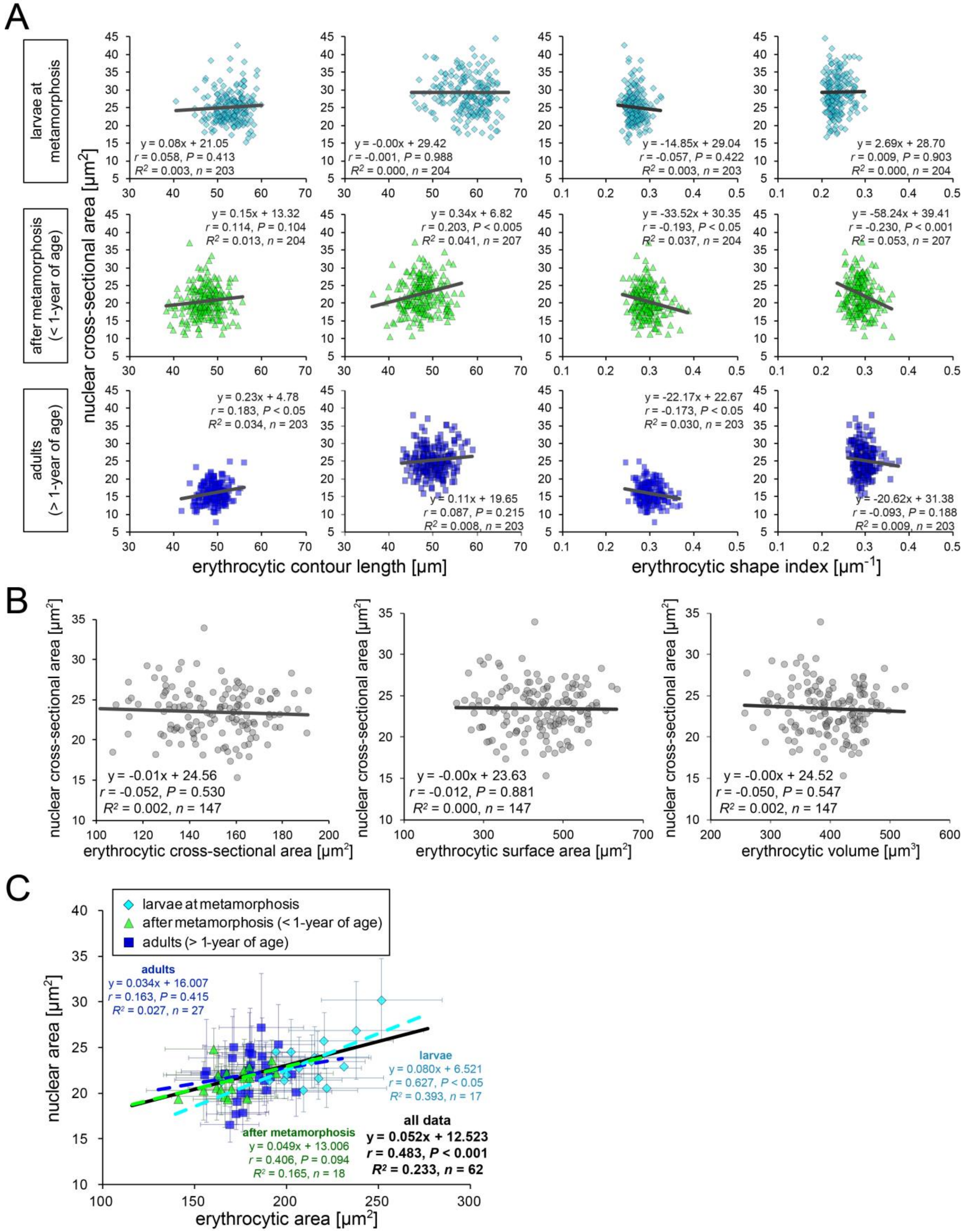

Supplementary Figure 2. (**A**) Measured nuclear cross-sectional areas were plotted against the erythrocytic contour lengths (left) or shape index (right), which is the ratio of the contour length to

cross-sectional area. Each symbol corresponds to data from each erythrocyte. The plots show the datasets from *X. laevis* individuals at the stages of larvae during metamorphosis (light blue), young adults (green), and fully grown adults (blue). The equation for the regression line, sample number (*n*), Pearson's correlation (*r*), *P* values, and the coefficient of determination (R^2) have been indicated in each plot. (**B**) Measured nuclear cross-sectional areas were plotted against the measured erythrocytic cross-sectional areas (left), estimated surface areas (middle), or volumes (right). The parameters were estimated from three-dimensional reconstruction. Each symbol corresponds to data from each erythrocyte. (**C**) Calculated mean nuclear cross-sectional areas were plotted against the mean erythrocytic cross-sectional area in a normal plot with a linear regression. Each symbol corresponds to the mean data (±SD) from each frog and each developmental stage. The equation for the regression line, *r*, *P* values, and R^2 have been indicated at each developmental stage (colored lines) and for the overall data (black). The datasets are identical to those in Figure 2E.

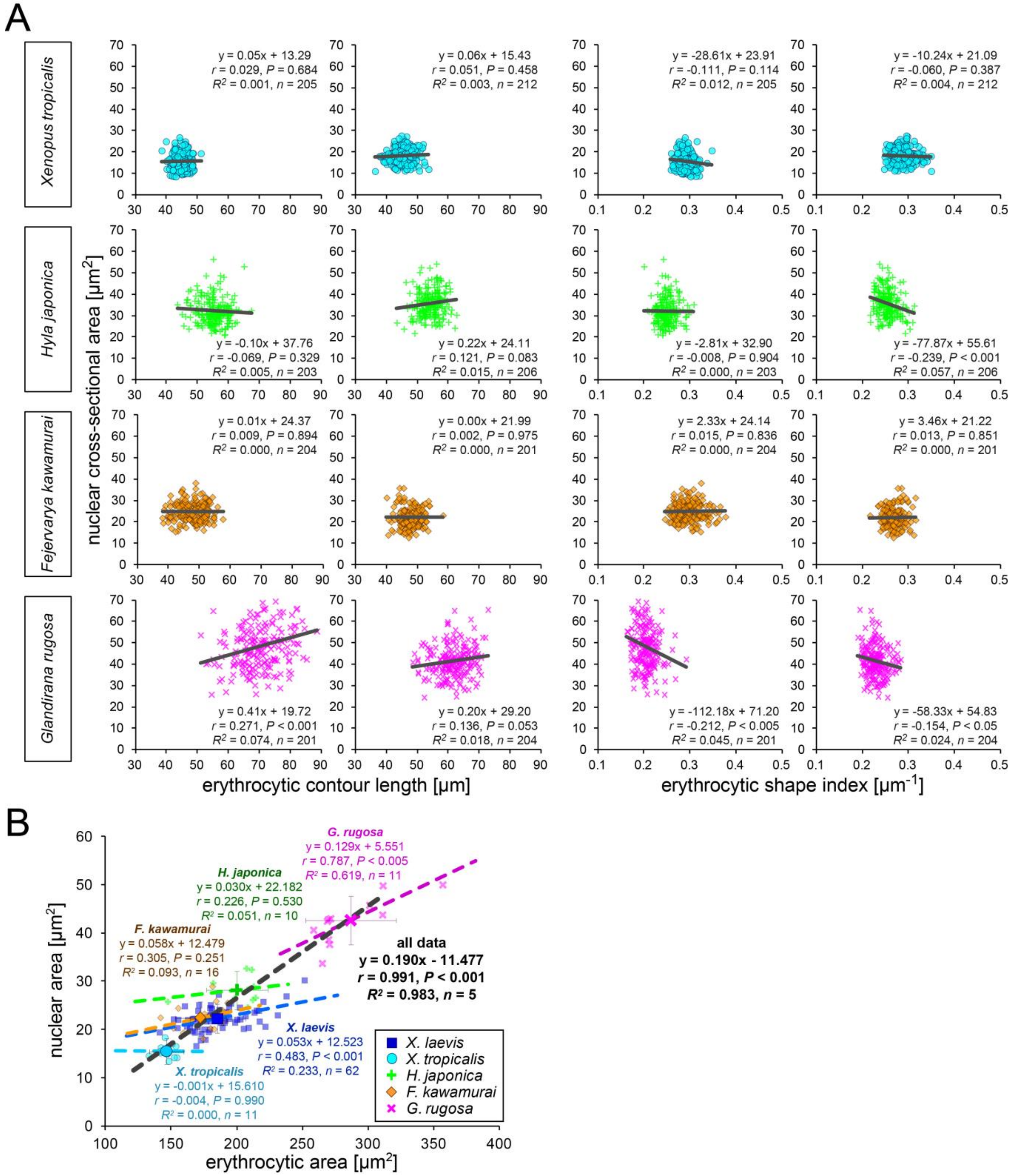

Supplementary Figure 3. (**A**) Measured nuclear cross-sectional areas were plotted against the erythrocytic contour lengths (left) or shape index (right), which is the ratio of the contour length to cross-sectional area, of adult frogs *X. tropicalis* (light blue), *H. japonica* (green), *F. kawamurai* (orange), and *G. rugosa* (pink). Each symbol corresponds to data from each erythrocyte. The equation for the regression line, sample number (*n*), Pearson's correlation (*r*), *P* values, and the coefficient of determination (R^2) have been indicated in each plot. (**B**) Calculated mean nuclear cross-

sectional areas were plotted against the mean erythrocytic cross-sectional area in a normal plot with a linear regression. The mean data from each frog (small symbols) and mean data from each species (big symbols; ±SD) have been shown, along with the equation for the regression line, *n*, *r*, *P* values, and R^2 have been indicated. The regression lines were calculated using mean data from each species. The datasets are identical to those in Figure 3A.

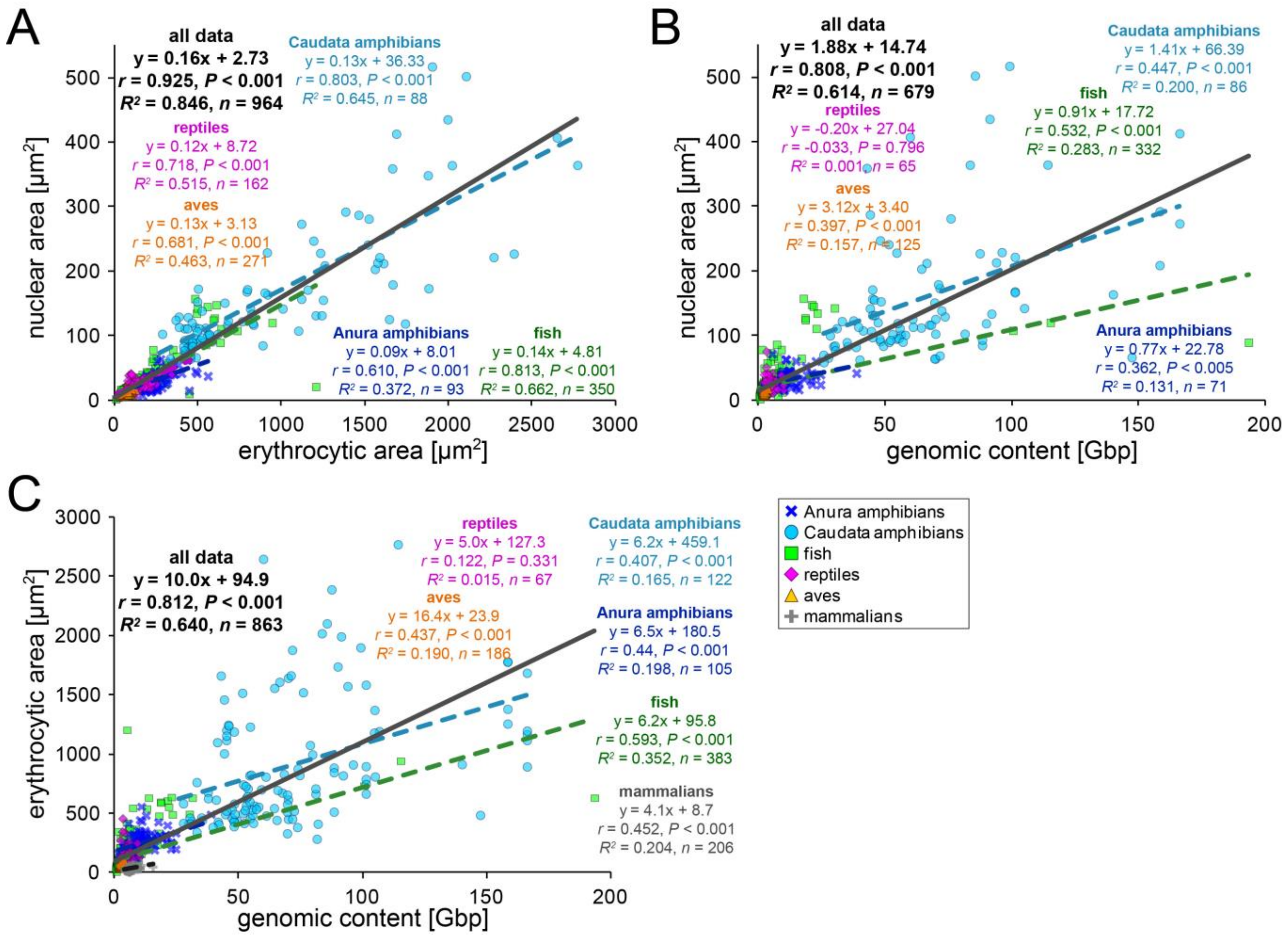

Supplementary Figure 4. (**A**) Collected nuclear cross-sectional areas were plotted against erythrocytic cross-sectional areas in a normal plot with linear regressions. (**B**) Collected nuclear and (**C**) erythrocytic cross-sectional areas were plotted against the genomic contents in a normal plot with linear regressions. Data from Anura amphibians (green), Caudata amphibians (green), fish (light blue), reptiles (purple), and aves (orange) have been shown. The equations for the regression line, sample number (*n*), Pearson's correlation (*r*), *P* values, and the coefficient of determination (R^2) have been indicated for datasets of each species class (colored broken lines) or all species together (grey line). The datasets are identical to those in 4A–4C.

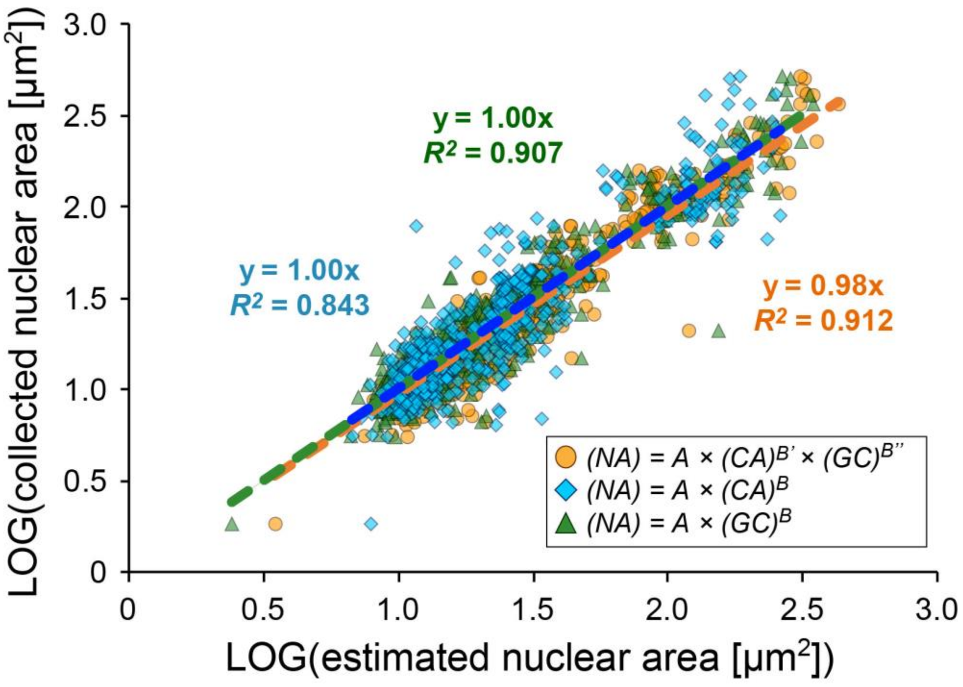

Supplementary Figure 5. Estimated nuclear cross-sectional areas were plotted against the collected nuclear cross-sectional areas in a log-log plot. Nuclear areas (orange symbols) were estimated for each species by fitting to equation combining two power-law parameters, which is $(NA) = A' \times (CA)^{B'} \times GC)^{B''}$. Nuclear areas (green or light blue symbols) were estimated for each species by fitting to an equation with single-powered parameters, such as $(NA) = A \times (CA)^B$ or $(NA) = A \times (GC)^B$, respectively. The regression lines and coefficients of determination (R^2) were shown for each fitting equation. Mean squared errors ($1/N \times \sum [(estimated\ nuclear\ area) - (collected\ nuclear\ area)]^2$) were 476 (in orange), 759 (in green), and 1384 (in blue).

Supplementary Tables

Supplementary Table 1. Size parameters of frog erythrocytes in our measurement. N.D.: not determined.

Supplementary Table 2. Size parameters of erythrocytes in our collected dataset. *n/a*: not available. Genomic content was calculated based on the C value listed in the Animal Genome Size Database (https://www.genomesize.com/).

Supplementary References

Andrews, C.B., Gregory, T.R. (2009). Genome size is inversely correlated with relative brain size in parrots and cockatoos. Genome. 52(3):261–267.

Arikan, H., Çiçek, K. (2014). Haematology of amphibians and reptiles: a review. North-West. J. Zool. 10(1):190–209.

Arizza, V., Russo, D., Marrone, F., Sacco, F., Arculeo, M. (2014). Morphological characterization of the blood cells in the endangered Sicilian endemic pond turtle, Emys trinacris (Testudines: Emydidae). Ital. J. Zool. 81(3):344–353.

Babaheydari, S.B., Keyvanshokooh, S., Dorafshan, S., Johari, S.A. (2016). Effects of tetraploidy induction on rainbow trout (Oncorhynchus mykiss, Walbaum, 1792) proteome at early stages of development. Comp. Biochem. Physiol. Part D Genome. Proteomics. 20:57–64.

Bogart, J.P., Klemens, M.W. (2008). Additional Distributional Records of Ambystoma Laterale, A. Jeffersonianum (Amphibia: Caudata) and Their Unisexual Kleptogens in Northeastern North America. American Museum Novitates 3627:1–58.

Bytyutskyy, D., Kholodnyy, V., Flajšhans, M. (2014). 3-D structure, volume, and DNA content of erythrocyte nuclei of polyploid fish. Cell Biol. Int. 38(6):708–715.

Cakmak, E., Cankiriligil, E.C., Düzgünes, Z.D., Ozel, O.T., Eroglu, O., Firidin, S. (2019). Triploid Black Sea Trout (Salmo labrax Pallas, 1814) Induced by Heat Shock and Evaluation of Triploidy with Different Techniques. Genet. Aquatic Organ. 3:1–7.

Calil, P.R., Puorto, G., Dunn, J.C., Chagas, C.R.F., Ramos, P.L. (2019). Molecular and morphological characterization of Hepatozoon spp. in Brazilian snakes. Amphibia-Reptilia 40(3): 337–347.

Casco-Robles, R.M., Watanabe, A., Eto, K., Takeshima, K., Obata, S., Kinoshita, T., Ariizumi, T., Nakatani, K., Nakada, T., Tsonis, P.A., Casco-Robles, M.M., Sakurai, K., Yahata, K., Maruo, F., Toyama, F., Chiba, C. (2018). Novel erythrocyte clumps revealed by an orphan gene Newtic1 in circulating blood and regenerating limbs of the adult newt. Sci. Rep. 8:7455.

Cook, C.A., Netherlands, E.C., Smit, N.J. (2016). Redescription, molecular characterisation and taxonomic re-evaluation of a unique African monitor lizard haemogregarine Karyolysus paradoxa (Dias, 1954) n. comb. (Karyolysidae). Parasit. Vectors 9:347.

Cui, L., Abbas, K., Yu, Y., Wang, W., Zhou, L., Zhou, X. (2013). First record of the natural occurrence of pentaploid loach, Misgurnus anguillicaudatus in Hubei Province, China. Folia. Zool. 62(1):14–18.

Deparis, P., Jaylet., A. (1984). The role of endoderm in blood cell ontogeny in the newt Pleurodeles waltl. J. Embryol. Exp. Morphol. 81:37–47.

Desser, S.S. (1978). Morphological, cytochemical, and biochemical observations on the blood of the tuatara, Sphenodon punctatus. N. Z. J. Zool. 5(3):503–508.

Dilrukshi, N.H.N., Jayasooriya, A.P., Prathapasinghe, G.A. (2019). Hematological parameters and morphological characteristics of blood cells in turtle and tortoise species within captivity in Sri Lanka. J. Adv. Vet. Anim. Res. 6(3):266–271.

Frýdlová, P., Hnízdo, J., Chylíková, L., Simková, O., Cikánová, V., Velenský, P., Frynta, D. (2013). Morphological characteristics of blood cells in monitor lizards: is erythrocyte size linked to actual body size? Integr. Zool. 8(1):39–45.

Fajer-Ávila, E.J., Guzman-Beltran, L., Zárate-Rodríguez, W.C., Río-Zaragoza, O.B. D., Almazan-Rueda, P. (2011). Pathology caused by adult Pseudochondracanthus diceraus (Copepoda: Chondracanthidae), a parasite of bullseye puffer fish Sphoeroides annulatus. Revista de Biologia Marina Y Oceanografia 46(3):293–302.

Gao, Z., Wang, W., Abbas, K., Zhou, X., Yang, Y., Diana, J.S., Wang, H., Wang, H., Li, Y., Sun, Y. (2007). Haematological characterization of loach Misgurnus anguillicaudatus: comparison among diploid, triploid and tetraploid specimens. Comp. Biochem. Physiol. A Mol. Integr. Physiol. 147(4):1001–1008.

Gregory, T.R. (2001). Coincidence, coevolution, or causation? DNA content, cell size, and the C-value enigma. Biol. Rev. 76(1):65–101.

Grenat, P., Salas, N., Pollo, F., Otero, M., Baraquet, M. (2018). Ulrich Sinsch3, Adolfo Martino1Naturally occurring triploids in contact zones between diploid/tetraploid Odontophrynus cordobae and O. americanus (Anura, Odontophrynidae). Amphibia-Reptilia 39:1–10.

Grosset, C., Wellehan, J.F.Jr, Owens, S.D., McGraw, S., Gaffney, P.M., Foley, J., Childress, A.L., Yun, S., Malm, K., Groff, J.M., Paul-Murphy, J., Weber, E.S.3rd. (2014). Intraerythrocytic iridovirus in central bearded dragons (Pogona vitticeps). J. Vet. Diagn. Invest. 26(3):354–364.

Gül, Ç., Tosunoğlu, M. (2011). Hematological reference intervals of four agamid lizard species from turkey (squamata: sauria: agamidae). Herpetzoa 24(1/2):51–59.

Hally, M.K., Rasch, E.M., Mainwaring, H.R., Bruce, R.C. (1986). Cytophotometric evidence of variation in genome size of desmognathine salamanders. Histochemistry. 85(3):185–192.

Horner, H.A., Macgregor, H.C. (1983). C value and cell volume: their significance in the evolution and development of amphibians. J. Cell Sci. 63:135–146.

Hu, T., Wu, C., Zhou, Y., Wang, S., Xiao, J., Wu, Y., Xu, K., Ren, L., Liu, O., Li, W., Wen, M., Tao, M., Qin, Q., Zhao, R., Luo, K., Liu, S. (2016). Chimeric Genes Revealed in the Polyploidy Fish Hybrids of Carassius cuvieri (Female) × Megalobrama amblycephala (Male). bioRxiv 082222.

Inumaru, M., Murata, K., Sato, Y. (2017). Prevalence of avian haemosporidia among injured wild birds in Tokyo and environs, Japan. Int. J. Parasitol. Parasites Wildl. 6(3):299–309.

Jalil, N., Alim, M.A., Abol-Munafi, A.B., Ariffin, N.A., Waiho, K. Sheriff, S.M. (2016). It is all in the Blood: Erythrocyte Characterization of Triploid and Diploid African Catfish, Clarias gariepinus. J. Fish. Aqua. Sci. 11:425–431.

Janiga, M., Haas, M., Kufelová, M. (2017). Age, sex and seasonal variation in the shape and size of erythrocytes of the alpine accentor, Prunella collaris (Passeriformes: Prunellidae). Eur. Zool. J. 84(1):583–590.

Jones, M.P. (2015). Avian hematology. Vet. Clin. North Am. Exot. Anim. Pract. 18(1):51–61.

Kim, G., Lee, M., Youn, S., Lee, E., Kwon, D., Shin, J., Lee, S., Lee, Y.S., Park, Y. (2018). Measurements of three-dimensional refractive index tomography and membrane deformability of live erythrocytes from Pelophylax nigromaculatus. Sci. Rep. 8(1):9192.

Lainson, R. De Souza, M.C., Franco, C.M. (2007). Natural and experimental infection of the lizard Ameiva ameiva with Hemolivia stellata (Adeleina: Haemogregarinidae) of the toad Bufo marinus. Parasite. 14(4):323–328.

Lisičić, D., Đikić, D., Benković, V., Horvat, A., Oršolić, K.N., Tadić, Z. (2013). Biochemical and hematological profiles of a wild population of the nose-horned viper Vipera ammodytes (Serpentes: Viperidae) during autumn, with a morphological assessment of blood cells. Zool. Stud. 52:11.

Lu, W., Liu, S., Long, Y., Tao, M., Zhang, C., Wang, J., Xiao, J., Chen, S., Liu, J., Liu, Y. (2009). Comparative study of erythrocytes of polyploid hybrids from various fish subfamily crossings. Cell Tissue Res. 336(1):159–163.

Mahony, M.J., Robinson, E.S. (1980). Polyploidy in the australian leptodactylid frog genus Neobatrachus. Chromosoma 81(2):199–212.

Maia, J.P., Harris, D.J., Carranza, S., Gómez-Díaz, E. (2014). A comparison of multiple methods for estimating parasitemia of hemogregarine hemoparasites (apicomplexa: adeleorina) and its application for studying infection in natural populations. PLoS One 9(4):e95010.

Majláthová, V., Majláth, I., Haklová, B., Hromada, M., Ekner, A., Antczak, M., Tryjanowski, P. (2010). Blood parasites in two co-existing species of lizards (Zootoca vivipara and Lacerta agilis). Parasitol Res. 107(5):1121–1127.

Martins, B.O., Franco-Belussi, L., Siqueira, M.S., Fernandes, C.E., Provete, D.B. (2021). The evolution of red blood cell shape in fishes. J. Evol. Biol. 34(3):537–548.

Mueller, R.L., Gregory, T.R., Gregory, S.M., Hsieh, A., Boore, J.L. (2008). Genome size, cell size, and the evolution of enucleated erythrocytes in attenuate salamanders. Zoology (Jena) 111(3):218–230.

Newman, C.E., Gregory, T.R., Austin, C.C. (2016). The dynamic evolutionary history of genome size in North American woodland salamanders. Genome 60(4):285–292.

Noleto, R.B., de Souza Fonseca Guimarães, F., Paludo, K.S., Vicari, M.R., Artoni, R.F., Cestari, M.M. (2009). Genome size evaluation in Tetraodontiform fishes from the Neotropical region. Mar. Biotechnol. 11(6):680–685.

Olmo, E., Morescalchi, A. (1978). Genome and cell sizes in frogs: A comparison with salamanders. Experientia 34:44–46.

Paperna, I., de Matos A.P.A. (1993). Erythrocytic viral infections of lizards and frogs: new hosts, geographical locations and description of the infection process. Ann. Parasitol. Hum. Comp. 68(1):11–23.

Pandian, T.J., Koteeswaran, R. (1999). Natural occurrence of monoploids and polyploids in the Indian catfish, Heteropneustes fossilis. Curr. Sci. 76(8):1134-1137.

Parto, P., Rastegar-Pouyani, N., Vaissi, S., Zarei, F., Karamiani, R. (2013). "Erythrocyte Sizes of Some Snake Species from West of Iran (Platyceps najadum najadum, Malpolon insignitus insignitus and Eirenis collaris) After Hibernation. World J. Zool. 8(3):324–327.

Rosset, S.D., Baldo, D., Lanzone, C., Basso, N.G. (2006). Review of the Geographic Distribution of Diploid and Tetraploid Populations of the Odontophrynus americanus Species Complex (Anura: Leptodactylidae). J. Herpet. 40(4):465–477.

Rossow, J.A., Hernandez, S.M., Sumner, S.M., Altman, B.R., Crider, C.G., Gammage, M.B., Segal, K.M., Yabsley, M.J. (2013). Haemogregarine infections of three species of aquatic freshwater turtles from two sites in Costa Rica. Int. J. Parasitol. Parasites. Wildl. 2:131–135.

Sari, I.G.A.A.R.P., Palupi, E.S. (2017). Hematological characteristic of the female Asian vine snake (Ahaetulla prasina Boie, 1827). UNEJ e-Proceeding, Sl:57–59.

Sayed, A.E.H., Kataoka, C., Oda, S., Kashiwada, S., Mitani, H. (2018). Sensitivity of medaka (Oryzias latipes) to 4-nonylphenol subacute exposure; erythrocyte alterations and apoptosis. Environ. Toxicol. Pharmacol. 58:98–104.

Seol, D.W., Im, S.Y., Hur, W.J., Park, M.O., Kim, D.S., Jo, J.Y., Park, I.S. (2008). Haematological parameters and respiratory function in diploid and triploid Far Eastern catfish (Silurus asotus). Genes Genomics 30(3):205–213.

Sloboda, M., Kamler, M., Bulantová, J., Votýpka, J., Modrý, D. (2007). A new species of hepatozoon (apicomplexa: adeleorina) from python regius (serpentes: pythonidae) and its experimental transmission by a mosquito vector. J. Parasitol. 93(5):1189–1198.

Small, S.A., Benfey, T.J. (1987). Cell size in triploid salmon. 241(3):339–342.

Stacy, N.I., Alleman, A.R, Sayler, K.A., (2011). Diagnostic Hematology of Reptiles, Clin. Lab. Med. 31(1):87–108.

Tosunoglu, M., Tok, C.V., Gul, C. (2005). Hematological Values in Hermann`s Tortoise (Testudo hermanni) and Spur-thigted Tortoise (Testudo greaca) from Thrace Region (Turkey). Int. J. Zool. Res. 1(1):11–14.

Uca, O., Arikan, H., Çiçek, K. (2017). Blood cell morphology of turkish gekkonid lizards (squamata: sauria: Gekkonidae, phyllodactylidae). Herpetozoa 30(1/2):29–37.

Ugurtas, I.H., Sevinc, M., Yildirimhan, H.S. (2003). Erythrocyte size and morphology of some tortoises and turtles from Turkey. Zool. Stud. 42(1):173–178.

Umezawa, T., Kato, A., Ogoshi, M., Ookata, K., Munakata, K., Yamamoto, Y., Islam, Z., Doi,.H,, Romero, M.F., Hirose, S. (2012). O_2-filled swimbladder employs monocarboxylate transporters for the generation of O_2 by lactate-induced root effect hemoglobin. PLoS One. 7(4):e34579.

Ursula, H., Rüdiger, K., Frank, M., Rinder, M. (2014). Blood parasites in reptiles imported to Germany. Parasitol Res. 113:4587–4599.

Vaissi, S., Fathipour, F., Ali Salamat, M., Parto, P., Sharifi. M. (2013). Variations in the size of erythrocytes and morphology of four lizard species (Laudakia nupta, Trapelus lessonae, Mabuya aurata and Ophisops elegans) from western Iran. Global Veterinaria 11(3):297–301.

van de Pol, I.L.E., Flik, G., Verberk, W.C.E.P. (2020). Triploidy in zebrafish larvae: Effects on gene expression, cell size and cell number, growth, development and swimming performance. PLoS ONE 15(3):e0229468.

Vilcins, I.M.E., Ujvari, B., Old, J.M., Deane, E. (2009). Molecular and Morphological Description of a Hepatozoon Species in Reptiles and Their Ticks in the Northern Territory, Australia. J. Parasitol. 95(2):434–442.

Villolobos, M., León, P., Sessions, S.K., Kezer, J. (1988). Enucleated Erythrocytes in Plethodontid Salamanders. Herpetologica, 44(2):243–250.

Wei, J., Li, Y.Y., Wei, L., Ding, G.H., Fan, X.L., Lin, Z.H. (2015). Evolution of erythrocyte morphology in amphibians (Amphibia: Anura). Zoologia 32(5):360–370.

Wolters, W. R., Chrisman, C. L. , Libey, G. S. (1982). Erythrocyte nuclear measurements of diploid and triploid channel catfish, Ictalurus punctatus (Rafinesque). J. Fish Biol. 20:253–258.

Zhu, D., Song, W., Yang, K., Cao, X., Gul, Y., Wang, W. (2012). Flow cytometric determination of genome size for eight commercially important fish species in China. In Vitro Cell Dev. Biol. Anim. 48(8):507–517.
